# Substrate and target selectivity of 4′-fluoroadenosine against viral and host polymerases

**DOI:** 10.1016/j.jbc.2026.113316

**Published:** 2026-07-06

**Authors:** Simon M. Walker, Arlo J. Loutan, Egor P. Tchesnokov, Dana Kocincova, Calvin J. Gordon, Ruby A. Escobedo, Nathaniel Jackson, Olivia A. Vogel, Kim Morsheimer, Suncheol Park, Anant Gharpure, Ixchel Urbano, Mina Heacock, Zhong Chen, Khushboo Pathak, Karen C. Wolff, Lauren Huerta, Malina A. Bakowski, Laura Riva, Anil K. Gupta, Chenguang Yu, Kalyan Das, Luis Martinez-Sobrido, Christopher F. Basler, Robert Davey, Ian A. Wilson, Andrew B. Ward, Sumit Chanda, Arnab K. Chatterjee, Matthias Götte

**Affiliations:** 1 Department of Medical Microbiology and Immunology, University of Alberta, Edmonton, Alberta, Canada; 2 Li Ka Shing Institute of Virology, University of Alberta, Edmonton, Alberta, Canada; 3 Texas Biomedical Research Institute, San Antonio, Texas, USA; 4 Department of Microbiology, Icahn School of Medicine at Mount Sinai, New York, New York, USA; 5 Department of Virology, Microbiology and Immunology, Boston University, Boston, Massachusetts, USA; 6 Department of Integrative Structural and Computational Biology, The Scripps Research Institute, La Jolla, California, USA; 7 Calibr-Skaggs Institute for Innovative Medicines, The Scripps Research Institute, La Jolla, California, USA; 8 Molecular Structural and Translational Virology, Department of Microbiology, Immunology and Transplantation, Rega Institute for Medical Research, KU Leuven, Leuven, Belgium

**Keywords:** antiviral drugs, enzymology, mechanism of action, nucleotide analogs, pandemic preparedness, RNA-dependent RNA polymerase, RNA viruses, cryo-EM

## Abstract

Developing safe and effective treatments against emerging RNA viruses is an important goal in pandemic preparedness efforts. 4′-fluorouridine (4′-FlU) is a broad-spectrum antiviral that was shown to inhibit viral RNA-dependent RNA polymerases (RdRps). Given its notable range of antiviral activity, this class of nucleoside analogs warrants further investigation. Here, we studied the antiviral activity and underlying mechanism of inhibition of 4′-fluoroadenosine (4′-FlA). Like 4′-FlU, 4′-FlA demonstrates a broad-spectrum of antiviral activity against eight prototypic viruses representing diverse families. Enzyme kinetics shows that the triphosphate (4′-fluoroadenosine triphosphate) is efficiently incorporated by viral RdRps. A cryo-EM structure of the RdRp of severe acute respiratory syndrome coronavirus 2 in complex with double-stranded RNA and the incorporated monophosphate (4'-fluoroadenosine monophosphate) characterizes interactions at the active site. The incorporated analog elicits heterogeneous inhibition patterns in primer extension reactions. In contrast, templates with embedded 4'-fluoroadenosine monophosphate inhibit incorporation of complementary uridine triphosphate (UTP) across the viral RdRps. However, incorporation of 4′-fluoroadenosine triphosphate is not limited to viral polymerases and likewise includes human mitochondrial RNA polymerase. These results demonstrate the general potential for 4′-fluorinated nucleotides as antiviral drugs and guide the development of more selective derivatives for medical use in appropriate settings.

Diverse positive- and negative-sense RNA viruses are associated with human diseases and have the potential to cause outbreaks, epidemics, or pandemics (1, 2). Severe acute respiratory syndrome coronavirus 2 (SARS-CoV-2) and the coronavirus disease 2019 pandemic provide a recent example and highlight the need for effective treatments during outbreaks. The viral RNA-dependent RNA polymerase (RdRp) is essential for viral replication and provides a logical target for the development of antiviral drugs (3). While structural features of these enzymes vary across viral families, the active site is generally conserved to accommodate nucleoside triphosphates (NTPs) (4, 5, 6). This feature provides opportunities to develop broad-spectrum antivirals that could be rapidly employed against emerging pathogens.

Nucleoside and nucleotide analogs (NAs) mimic natural NTPs and commonly inhibit viral RNA synthesis once incorporated into the growing RNA strand (4, 7). Remdesivir (RDV), a 1′-cyano modified *C*-linked adenosine monophosphate prodrug, interferes with RNA synthesis by SARS-CoV-2 RdRp and has been approved for the treatment of coronavirus disease 2019 (8, 9). RDV shows antiviral activity against a broad spectrum of virus families, including the *Corona-*, *Picorna-*, *Flavi-*, *Filo-*, *Paramyxo-*, and *Pneumoviridae* (10, 11, 12, 13, 14). RDV is less active or not active against several segmented negative-sense viruses, such as members of the *Nairoviridae* (Crimean-Congo hemorrhagic fever (CCHFV), *Arenaviridae*, and *Orthomyxoviridae* (influenza viruses) (10, 11). Molnupiravir, a cytidine analog and prodrug of β-D-N^4^-hydroxycytidine (NHC), has also demonstrated a broad range of activity, including several segmented negative-sense RNA viruses (15, 16, 17, 18, 19, 20, 21, 22, 23, 24). The investigational 4′-fluorouridine (4′-FlU) shows a similar profile (25, 26, 27, 28, 29, 30, 31, 32, 33). While molnupiravir acts as a mutagenic nucleotide, RDV and 4′-FlU inhibit viral RNA synthesis (34).

Data that identify the biochemical attributes underlying the broad-spectrum activity of 4′-FlU remain elusive. Previous work has demonstrated that the active triphosphate form, 4′-FlU-TP, is used as a substrate by the viral RdRp of respiratory syncytial virus (RSV), SARS-CoV-2, and influenza A (FluA) (29, 30). For both RSV and FluA RdRp, the natural counterpart UTP outcompetes 4′-FlU-TP approximately 4-fold. Once incorporated, 4′-FlU displays heterogeneous effects on primer-strand extension. Against RSV RdRp, inhibition at the site of incorporation “i” and further downstream point to contributions from immediate and delayed chain-termination, respectively (30). For SARS-CoV-2 RdRp, a single incorporation of 4′-FlU still generates the full-length product (30). In the case of FluA RdRp, there is evidence for immediate chain-termination (29). Overall, the mechanism of action of 4′-FlU appears heterogeneous and dependent on the nature of the viral RdRp.

Collectively, these studies on 4′-FlU raise several important questions. Does the 4′-fluoro modification also mediate antiviral effects in a different base context? How specific is the inhibition of viral polymerases? Is there a common underlying mechanism of inhibition against diverse polymerase targets? To address these questions, we synthesized a purine derivative of 4′-FlU, 4′-fluoroadenosine (4′-FlA) (Fig. 1*A*), and show that the spectrum of antiviral activity is comparable to that of 4′-FlU. The active form, 4′-FlA-TP, is efficiently incorporated by an array of corresponding viral RNA polymerases. We determined a cryo-EM structure of SARS-CoV-2 RdRp with the NA to identify its binding pose at the active site. Comparison with other viral polymerase structures demonstrates broad-spectrum antiviral activity. Several host polymerases also use 4′-FlA-TP as a substrate, which needs to be addressed in promising drug development programs.

**Figure 1:**
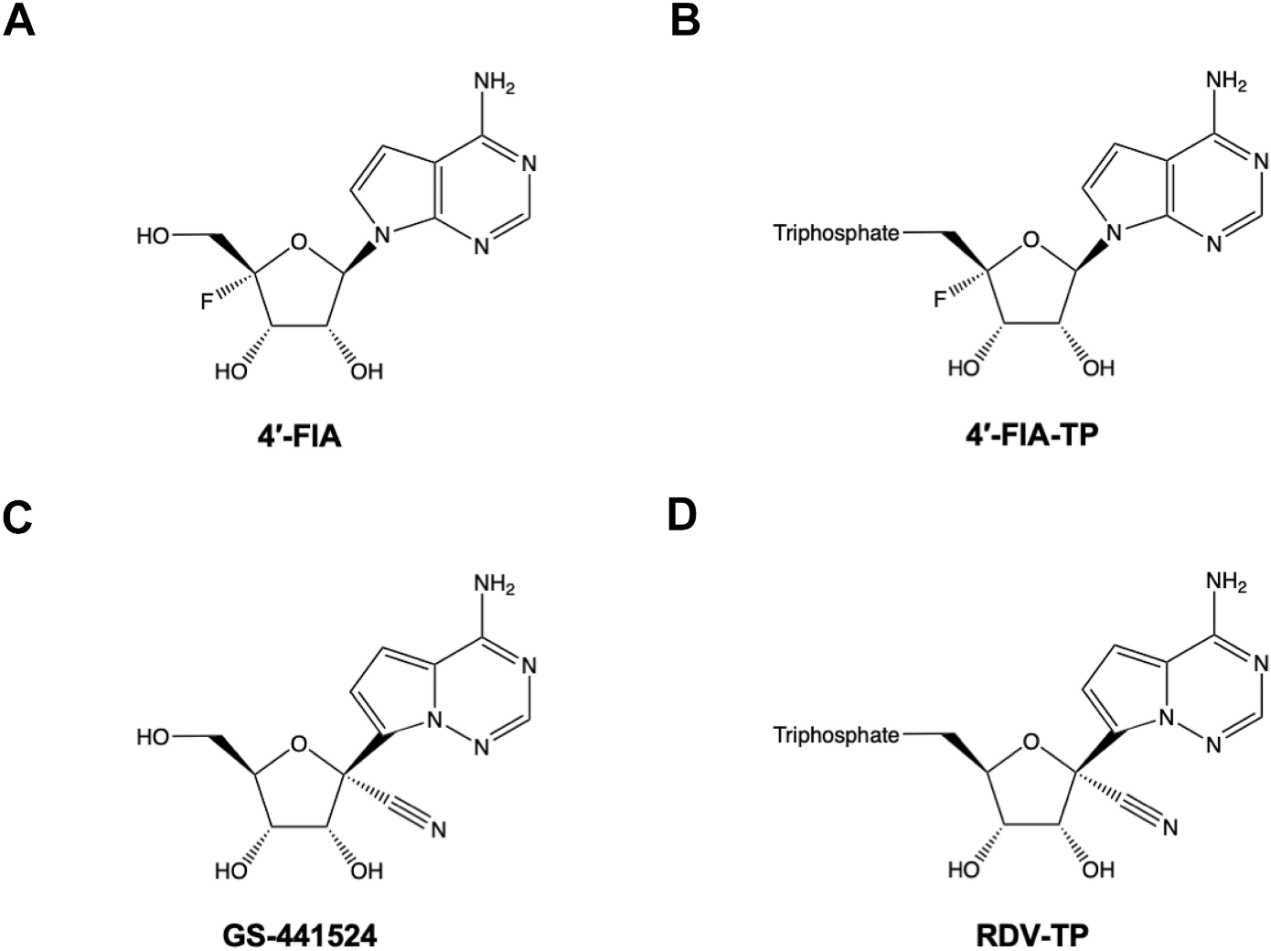
**Chemical structures of nucleoside and nucleotide analogs investigated in this study.** 4′-fluoroadenosine or 4′-FlA (*A*), 4′-fluoroadenosine triphosphate or 4′-FlA-TP (*B*), GS-441524 (*C*), and remdesivir triphosphate or RDV-TP (*D*).

## Results

### Experimental strategy

The main objective of this work was to evaluate the efficacy and selectivity of 4′-FlA against RNA viruses that represent families of epidemic or pandemic potential. Prototypic viruses from diverse families were considered: *Flaviviridae* (DENV-2), *Coronaviridae* (SARS-CoV-2), *Picornaviridae* (human rhinovirus [HRV-16]), *Pneumoviridae* (RSV), *Filoviridae* (Ebola virus [EBOV]), *Nairoviridae* (CCHFV), and *Orthomyxoviridae* (FluA and FluB). Biochemical enzymatic assays and structural evaluation were used to assess 4′-FlA-TP (Fig. 1*B*) incorporation and the mechanism of inhibition of RNA synthesis. RDV-TP (Fig. 1*D*) was used for comparative purposes, and host polymerases were considered to evaluate the potential for off-target effects.

### Antiviral activity and cytotoxicity of 4′-FlA

4′-FlA demonstrates antiviral effects against a panel of prototypic viruses (Table 1). Antiviral efficacy by increasing effective inhibitory concentrations (EC_50_) values follows the order DENV-2 (0.016 μM) > RSV (0.061 μM) > FluA (0.067 μM) > SARS-CoV-2 (0.071 μM) > CCHFV (0.076 μM) > FluB (<0.1 μM) > HRV-16 (0.14 μM) > EBOV (0.22 μM). Against the EBOV minigenome, 4′-FlA similarly demonstrates antiviral activity (0.17 μM). The 50% cytotoxic concentration (CC_50_) for 4′-FlA varies due to the cell types employed, ranging from 1.0 to >50 μM, and corresponding selectivity index (SI) values ranging from ∼10 to >100, indicating the observed antiviral activities are predominantly driven by polymerase inhibition.

**Table 1:** Antiviral activity and cytotoxicity of GS-441524 and 4′-FlA^a^

Virus	Cell line	GS-441524			4′-FlA		
		EC_50_ (μM)	CC_50_ (μM)	Selectivity index (SI)^b^	EC_50_ (μM)	CC_50_ (μM)	Selectivity index (SI)
DENV-2	HepG2	4.9 ± 1.7 9.46^c^	> 99	>20	0.016 ± 0.004	3.3 ± 0.8	210
HRV-16	H1 HeLa	14.6 ± 6.9 (*n* = 2)	> 99	>6.8	0.14 ± 0.16 (*n* = 2)	2.1 ± 0.7	15
SARS-CoV-2	HeLa-ACE2	8.7 ± 0.4 0.28–1.65^d^	> 99	>11	0.071 ± 0.010	1.00 ± 0.03	14
RSV	Hep2	0.89 ± 0.20 0.63^e^	> 99	>110	0.061 ± 0.027	3.2 ± 1.0	52
EBOV	HeLa	0.17 (*n* = 1) 0.51 ± 0.04^f^ 1.0–3.1^e^	8.02 ± 0.04^f^ > 49^e^	16 16–50^e^	0.22 ± 0.06 (*n* = 2) 0.17 ± 0.12^f^	5.5 ± 1.1 1.6 ± 0.1^f^	25 9.4
CCHFV	Vero-E6	No inhibition^e^			0.076 (*n* = 1)	>50	>660
FluA	A549	>10 (*n* = 2) 27.9^c^	>99 >30^c^	>9.9 >1.1^c^	0.067 ± 0.008	2.8 ± 0.3	42
FluB	MDCK	-	-	-	< 0.1 (*n* = 1)	30	300

DENV-2, dengue virus 2; HRV-16, human rhinovirus 16; SARS-CoV-2, severe acute respiratory syndrome coronavirus 2; RSV, respiratory syncytial virus; EBOV, Ebola virus; CCHFV, Crimean-Congo hemorrhagic fever virus; FluA, influenza A virus; FluB, influenza B virus; 4′-FlA, 4′-fluoroadenosine.

a The antiviral activity of GS-441524 and 4′-FlA was evaluated against a panel of viruses in different cell types as described in Materials and Methods to determine the half-maximum effective concentration (EC_50_) and half-maximal cytotoxic concentration (CC_50_). All data are at least *n ≥ 3* replicates (unless otherwise indicated) and reported as the average ± SD.

b Selectivity index = CC_50_/EC_50_.

f Half maximal inhibitory concentration (IC_50_) of 4′-FlA determined in the EBOV minigenome assay. Data are at least *n = 2* replicates and reported as the average ± SD.

c Data from Cho *et al.,* 2012 (35).

d Data from Pruijssers *et al*., 2020 (13).

e Data from Lo *et al.,* 2017 (11).

GS-441524, the parent nucleoside of RDV, was also evaluated for comparative purposes (Fig. 1*C*). In cell-based assays, EC_50_ values for RDV and GS-441524 against several of the viruses discussed here have been previously determined (10, 11, 12, 13, 14, 35). EC_50_ values for RDV and GS-441524 are in the sub-to-low micromolar range across many viral families, though the prodrug has greater activity, likely due to higher levels of active 5′-triphosphate metabolite formation within the cell (13). Here, we determined EC_50_ values for GS-441524 against DENV-2, HRV-16, SARS-CoV-2, RSV, and EBOV (Table 1). Our observed EC_50_ values for GS-441524 are largely consistent with previous reports, as indicated in the Table. Taken together, although 4′-FlA demonstrates a broader spectrum of antiviral activities as compared to RDV, the lower CC_50_ values are consistent with potential off-target effects.

### Incorporation of 4′-FlA-TP by representative viral polymerases

4′-FlA-TP, the active triphosphate form of 4′-FlA, is anticipated to compete with natural ATP pools for incorporation by the viral polymerase. Furthermore, previous studies have demonstrated that efficient incorporation of an NA is an important parameter to assess the potential for antiviral activity (36, 37, 38). We compared the incorporation efficiency of ATP and 4′-FlA-TP with purified recombinant polymerases and polymerase complexes from the viruses for which 4′-FlA was tested (Table 2 and Table S1). For DENV-2, HRV-16, SARS-CoV-2, RSV, EBOV, CCHFV, and FluB, *in vitro* enzyme RNA synthesis was evaluated using short model primer/templates as previously described (12, 36, 37, 39, 40) (Fig. S1). FluA RdRp RNA synthesis activity requires the use of a genomic vRNA template, utilizing a radiolabeled 13-nt 5′-methyl-7-guanosine capped primer (Fig. S2). Steady-state kinetic parameters *V*_max_ and *K*_m_ were measured to determine substrate selectivity, defined as the ratio of the incorporation efficiency of natural nucleotide over the NA. A selectivity value below one suggests that the analog is more efficiently incorporated than the natural nucleotide. The relative efficiency of 4′-FlA-TP incorporation follows the order HRV-16 (0.48) > SARS-CoV-2 (0.99) > CCHFV (1.4) ∼ FluB (1.4) > EBOV (4.2) > RSV (7.1) > DENV-2 (7.6) (Table 2). Using the 13-nt capped primer system, FluA and FluB demonstrate selectivity values of 1.5 and 0.52, respectively (Tables 2 and S2). The low selectivity values for CCHFV, FluA, and FluB RdRp are a distinct property of 4′-FlA-TP in comparison to RDV-TP. Collectively, these data suggest that the 4′-fluoro modification is well tolerated across all viral polymerases tested and that 4′-FlA-TP can efficiently compete with the natural nucleotide ATP.

**Table 2:** Incorporation of RDV-TP and 4′-FlA-TP by selected viral RdRp enzymes

RNA Sense	Family	Virus	RDV-TP	4′-FlA-TP
			Selectivity^a^ (fold)	
Positive ssRNA	*Flaviviridae*	DENV-2	11.1^b^	7.6
	*Picornaviridae*	HRV-16	1.03^c^	0.48
	*Coronaviridae*	SARS-CoV-2	0.28^d^	0.99
Nonsegmented negative ssRNA	*Pneumoviridae*	RSV	2.7^e^	7.1
	*Filoviridae*	EBOV	4.0^d^	4.2
Segmented negative ssRNA	*Nairoviridae*	CCHFV	41^f^	1.4
	*Orthomyxoviridae*	FluB	68^f^	1.4
Segmented negative ssRNA	*Orthomyxoviridae*	FluA^g^	1800	1.5
		FluB^g^	1400	0.52

DENV-2, dengue virus 2; HRV-16, human rhinovirus 16; SARS-CoV-2, severe acute respiratory syndrome coronavirus 2; RSV, respiratory syncytial virus; EBOV, Ebola virus; CCHFV, Crimean-Congo hemorrhagic fever virus; FluA, influenza A virus; FluB, influenza B virus; RDV-TP, remdesivir triphosphate; 4′-FlA-TP, 4′-fluoroadenosine triphosphate.

a Selectivity of a viral polymerase for a nucleotide analog is calculated as the ratio of the *V*_max_/*K*_m_ values for ATP and the analog, respectively.

b Data from Walker *et al*., 2026 (12).

c Data from Gordon *et al.,* 2024 (36).

d Data from Gordon *et al.,* 2020 (41).

e Data from Tchesnokov *et al.,* 2019 (40).

f Data from Gordon *et al.,* 2022 (37).

g Selectivity values generated for influenza A and FluB RdRp at the bottom of the table were performed using a 13-nt labelled primer/template system (see Fig. S2 for assay details). All other values in the table were generated using the 4-nt primer/template system (see Fig. S1 for assay details).

### Heterogeneous effects of 4′-FlA-TP in the primer-strand

**Figure 2:**
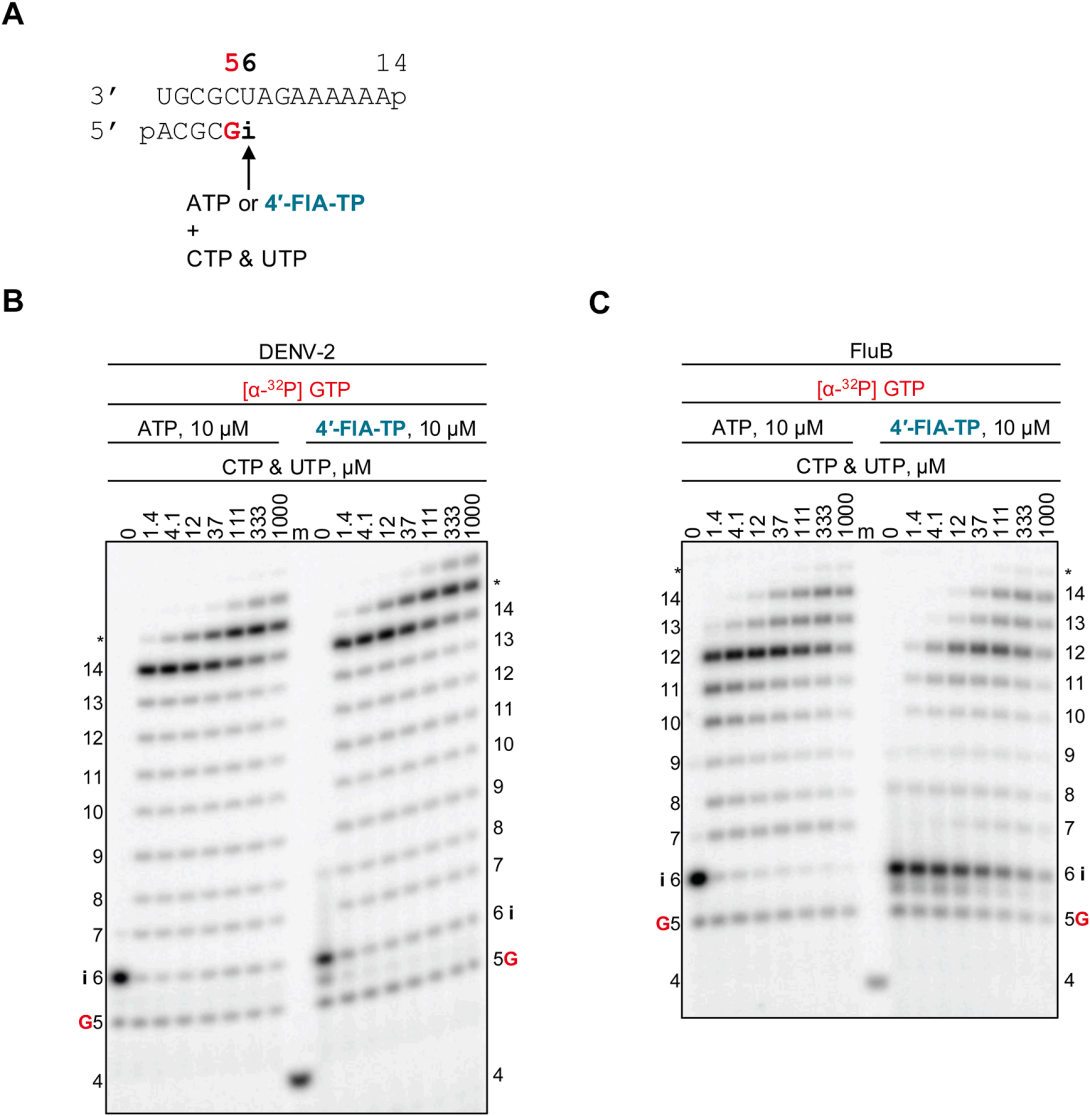
**DENV-2 and FluB RdRp-catalyzed RNA synthesis and inhibition patterns following a single incorporation of ATP or 4′-FlA-TP as a function of nucleotide concentration.***A,* RNA primer/template supporting a single incorporation of ATP or 4′-FlA-TP at position 6 (“i”). G indicates incorporation of [α-^32^P]-GTP at position 5. Migration pattern of RNA products resulting from *B,* DENV-2 RdRp- and *C,* FluB RdRp-catalyzed RNA extension following incorporation of ATP or 4′-FlA-TP at position 6 (“i”) at increasing CTP and UTP concentrations. A 5′-^32^P-labelled 4-nt primer (4) serves as a size marker (m). RNA products formed at and beyond the asterisk may be a result of sequence-dependent slippage events or RdRp nucleotide transferase activity. 4′-FlA-TP, 4′-fluoroadenosine triphosphate; DENV-2, dengue virus 2; FluB, influenza B virus; RdRp, RNA-dependent RNA polymerase.

**Figure 3:**
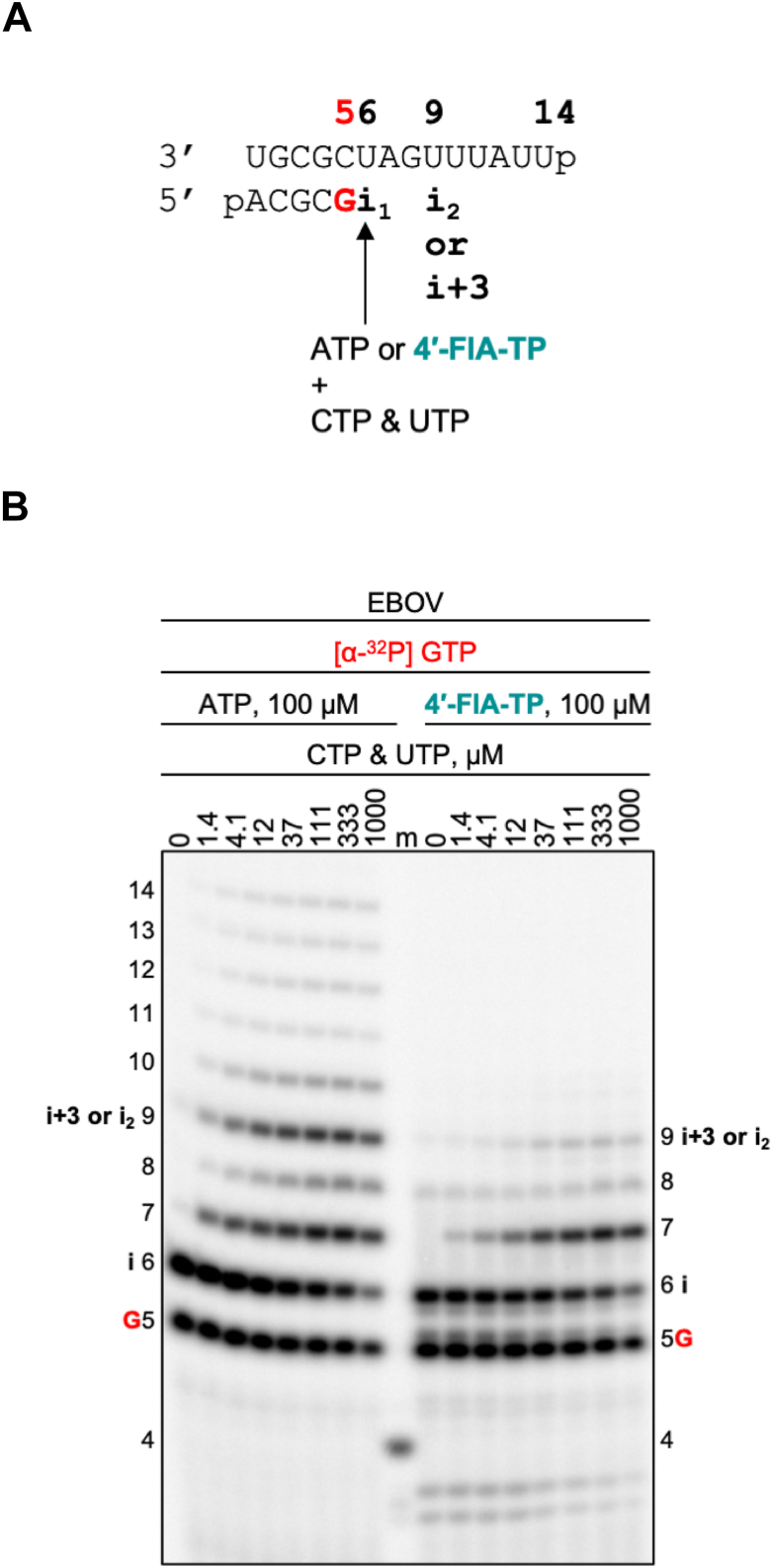
**EBOV RdRp-catalyzed RNA synthesis and inhibition patterns following multiple incorporations of ATP or 4′-FlA-TP as a function of nucleotide concentration.***A,* RNA primer/template supporting a first and second incorporation of ATP or 4′-FlA-TP at position 6 (“i”) and position 9 (“i_2_”). G indicates incorporation of [α-^32^P]-GTP at position 5. *B,* Migration pattern of RNA products resulting from EBOV RdRp-catalyzed RNA extension following multiple incorporations of ATP or 4′-FlA-TP at increasing CTP and UTP concentrations. A 5′-^32^P-labelled 4-nt primer (4) serves as a size marker (m). Multiple 4′-FlA-TP incorporations do not appear to allow production of full-template length product. EBOV, Ebola virus; RdRp, RNA-dependent RNA polymerase; 4′-FlA-TP, 4′-fluoroadenosine triphosphate.

Incorporation of an NA into the growing RNA primer-strand is commonly required for downstream inhibition. We evaluated the patterns of RNA synthesis following the incorporation of a single 4′-FlA-TP (Fig. 2). For DENV-2 RdRp, 4′-FlA-TP does not appear to inhibit primer extension (Fig. 2*B*). Similarly, 4′-FlA-TP demonstrates no inhibitory effect during primer-strand synthesis against HRV-16 and SARS-CoV-2 (Fig. S3). In contrast, 4′-FlA-TP incorporation elicits immediate chain termination against RdRp from FluB and CCHFV (Figs. 2*C* and S4). 4′-FlA-TP incorporation by RSV RdRp appears to elicit an inhibitory effect downstream at position “i + 3” (Fig. S5*A*). A template allowing for a second 4′-FlA-TP incorporation enhances the inhibitory effect at this position (Fig. S5*B*), which is also observed for EBOV (Fig. 3). Using the 13-nt radiolabeled capped primer and vRNA template, 4′-FlA-TP incorporation results in immediate chain termination against both influenza RdRp (Fig. S6). A second inhibitory site at position “i + 2” can also be seen, which may be due to a second incorporation event of 4′-FlA-TP. Notably, against all viral RdRp, longer RNA products past the site of a single incorporation of 4′-FlA-TP can be generated. As observed with influenza, RSV, and EBOV RdRps, inhibition due to multiple incorporations appears more absolute.

To translate these findings into quantitative terms, we determined the inhibitory efficiency of subsequent nucleotide incorporation following the incorporation of 4′-FlA-TP. We determined the kinetic parameters of UTP incorporation at position “i + 1” with AMP- and 4′-FlA-MP-terminated primer strands to determine inhibition values (Fig. S7 and Table S3). For the positive-sense RNA viruses, the extension of 4′-FlA-terminated primers is only slightly impaired, 3- to 5-fold, compared to AMP. Against RSV and EBOV, the 4′-FlA-terminated primer displays 13- and 8-fold inhibition. For CCHFV, UTP incorporation with 4′-FlA-MP-terminated primers is inhibited roughly 33-fold. Against FluB, the extension of 4′-FlA-MP-terminated primer requires a marked 710-fold increase in UTP concentration. Thus, 4′-FlA-TP exhibits variable inhibition on subsequent nucleotide incorporation. Overall, inhibition within the primer-strand by a single incorporated 4′-FlA-TP appears heterogeneous and not absolute.

### Template-dependent inhibition by an embedded 4′-FlA residue

**Figure 4:**
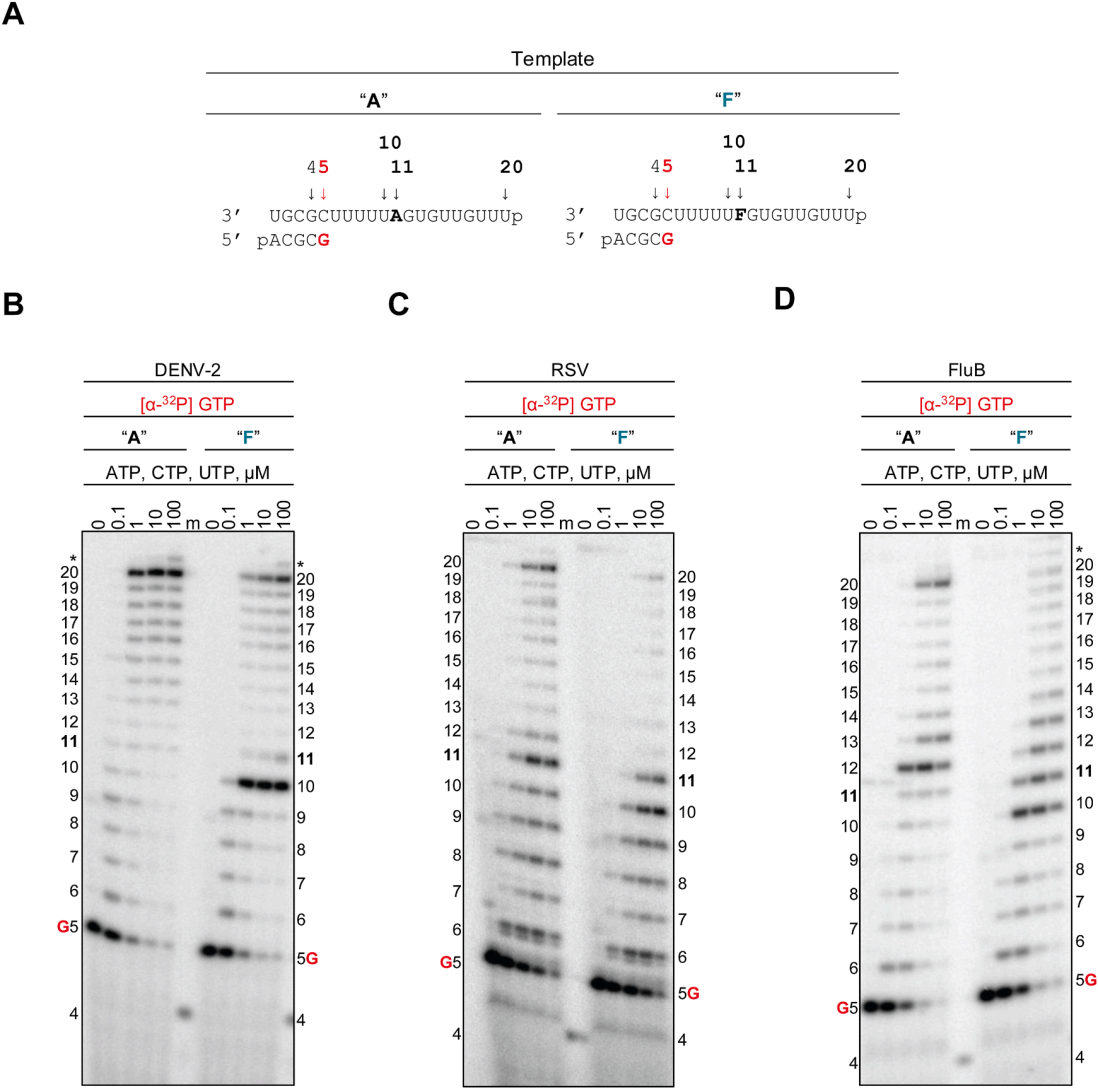
**Template-dependent inhibition of DENV-2, RSV, and FluB RdRp by a single embedded 4′-FlA residue at position 11.***A,* RNA primer/template with an embedded adenosine (Template “A”, *left*) and (Template “F”, *right*) at position 11. G indicates incorporation of [α-^32^P]-GTP at position 5. Migration pattern of RNA products resulting from *B,* DENV-2 RdRp-, *C,* RSV RdRp-, and *D,* FluB RdRp-catalyzed RNA synthesis supplemented with increasing NTP concentrations using Template “A” (*left*) and Template “F” (*right*). A 5′-^32^P-labelled 4-nt primer (4) serves as a size marker (m). RNA products formed at and beyond the asterisk may be a result of sequence-dependent slippage events or RdRp nucleotide transferase activity. 4′-FlA, 4′-fluoroadenosine; DENV-2, dengue virus 2; RSV, respiratory syncytial virus; FluB, influenza B virus; RdRp, RNA-dependent RNA polymerase; NTP, nucleoside triphosphate.

Efficient incorporation of 4′-FlA-TP into the growing primer-strand and the ability of the viral RdRp to overcome the inhibitory effects provide the opportunity for the RdRp to synthesize full-length RNA copies. Thus, newly synthesized RNA copies could contain embedded analog residues that affect RNA synthesis when used as a templating-base. This mechanism of inhibition has been studied for RDV against numerous viral RdRps and provides a unifying mechanism of inhibition that describes its broad-spectrum antiviral activity (12, 36, 37, 41). Here, we designed RNA templates with a single AMP or 4′-FlA-MP at position 11 (Figs. 4*A* and S8*A*). For all viral RdRp, low micromolar concentrations (1–10 μM) of NTPs are sufficient to generate full-length product on Template “A” (Figs. 4, *B*–*D* and S8, *B*–*D*). Comparatively, on Template “F”, inhibitory sites prior to the embedded 4′-FlA-MP and a corresponding decrease in full-template length RNA are seen. This inhibitory effect is maintained despite increasing NTP concentrations, though an increase in full-length product is observed. Further evaluation demonstrated that UTP incorporation opposite a template-embedded 4′-FlA-MP is most efficient among all NTPs; however, this incorporation is reduced relative to a template-embedded AMP at the equivalent position (Fig. S9). Thus, 4′-FlA impairs the incorporation of incoming UTP when it is embedded in the template, providing a unifying mechanism of action that helps to explain the broad spectrum of antiviral activity of 4′-FlA.

### Cryo-EM structure of incorporated 4′-FlA-MP by SARS-CoV-2 RdRp

**Figure 5:**
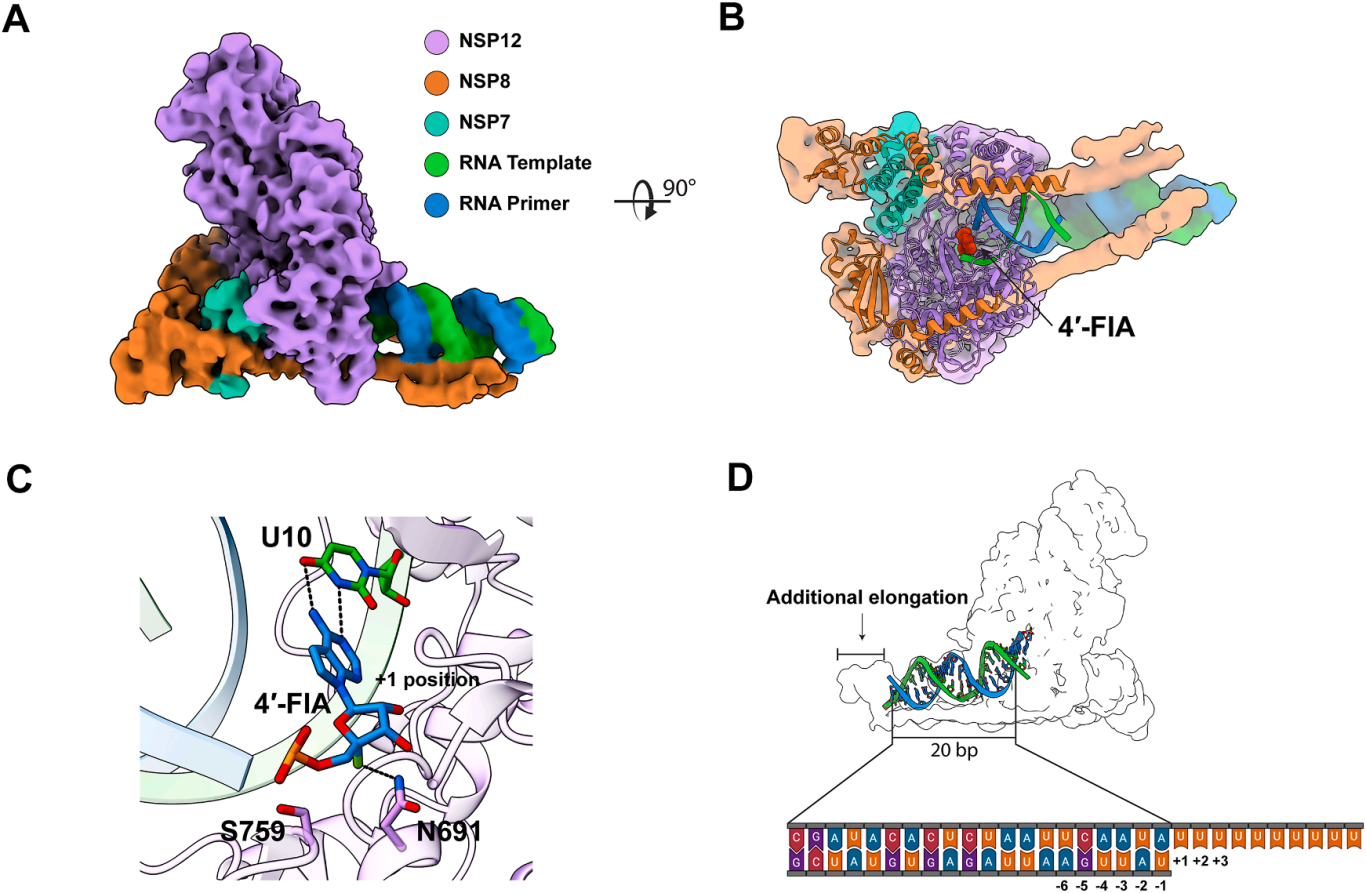
**Cryo-EM structure of the SARS-CoV-2 RdRp complex bound to RNA and 4′-FlA.***A and B,* Cryo-EM density map of the SARS-CoV-2 RdRp complex, low-pass filtered to 5 Å. The *left panel* (*A*) shows a *side* view, and the *right panel* (*B*) shows a *bottom* view of the complex. The atomic model is displayed as a cartoon representation fitted within the transparent surface map. 4′-FlA is shown as a red sphere model at the active site. *C,* close-up view of the RdRp active site showing 4′-FlA at the +1 position, base-paired with the template strand nucleotide U10. Hydrogen bonds between 4′-FlA and U10 are indicated by *dashed lines*. Residue N691 of NSP12, located within 3.5 Å of 4′-FlA, is indicated by *dashed lines* and labelled. *D,* schematic representation of the RNA scaffold used for cryo-EM structure determination. The 20 bp double-stranded region is indicated. Additional elongation beyond the designed dsRNA region is shown, with overhanging uracil residues on the template strand. Nucleotide positions are labelled (−6 to −1 and +1 to +3). SARS-CoV-2, severe acute respiratory syndrome coronavirus 2; 4′-FlA, 4′-fluoroadenosine.

We determined the cryo-EM structure of the SARS-CoV-2 RdRp complex comprising nsp12, nsp8, and nsp7 in complex with RNA and incorporated 4′-FlA-MP at an overall resolution of 3.4 Å (Fig. 5 and Table S4). Structural analysis reveals that 4′-FlA-MP occupies the +1 or “i” active site position, equivalent to the binding site previously described for RDV in the SARS-CoV-2 RdRp (42) (Figs. 5, *B*, *C*, and S10, *A*, *B*). At this position, 4′-FlA forms a base pair with the U10 nucleotide of the template strand (Fig. 5*C*). Although the precise orientation of the 4′-fluorine modification on 4′-FlA cannot be unambiguously defined due to limited local resolution, it is positioned in close proximity to residues S759 and N691 of nsp12 that are also involved in RDV-TP binding (43). Comparisons to structures of DENV-2 RdRp (PDB: 7XD8) (44) and FluB RdRp (PDB: 6QCT) (45) suggest that 4′-FlA could bind in a similar pose at their respective active sites, further highlighting the potential for broad-spectrum activity (Fig. S10, *E* and *F*).

In our cryo-EM structure, the RNA density observed in the RdRp-bound complex extends beyond the 20-base-pair double-stranded region used for structure determination, indicating that additional elongation had occurred (Fig. 5*D*). The reaction between the overhanging uracil residues on the template strand and 4′-FlA incubated with the RNA demonstrates that 4′-FlA supports continued primer extension activity. In contrast to RDV against SARS-CoV-2, which exhibits delayed-chain termination due to a clash between the 1′-cyano modification and S861 (41, 46, 47, 48), 4′-FlA appears to mediate a distinct mechanism in the primer-strand. To further evaluate potential steric conflicts introduced by the 4′-fluoro modification at each RNA position, we performed structural modelling using the previously determined 2.5 Å resolution structure (PDB: 7BV2) (42) (Fig. S10, *G* and *H*). In the context of the primer-strand, the 4′-fluorine atom is positioned within 3 Å of the main chain atom of C813 and the side chain of D865 at the −2 and −3 positions, respectively, indicating a potential for steric clash. In contrast, when the 4′-fluoro modification is modelled into the template strand, the fluorine atom falls within 3 Å of the main chain atoms of G683, G590, and S592, as well as the side chains of N543 and F594, suggesting a higher likelihood of steric conflict at these positions. Taken together, these observations indicate that the presence of the 4′-fluoro modification within the template strand of SARS-CoV-2 RdRp could interfere with polymerase activity.

### Incorporation of NAs by host enzymes

Incorporation of NAs by host polymerases can be associated with cytotoxic effects (49, 50, 51, 52). Here, we examined the use of 4′-FlA-TP as a substrate for h-mtRNAP, comparing selectivity with approved NAs (Figs. S11, S12, Table S5). Included were RDV-TP, NHC-TP, and sofosbuvir (SOF), a 2′-*α*-fluoro, 2′-*β*-methyluridine monophosphate prodrug approved for the treatment of hepatitis C virus infection (53, 54). Our selectivity values of RDV-TP, NHC-TP, and SOF-TP for h-mtRNAP are consistent with published data (Table 3). RDV-TP is incorporated 2200-fold less efficiently than ATP, consistent with previous reports (40). These data demonstrate that RDV-TP is a poor substrate for h-mtRNAP, indicating low potential cytotoxicity and corroborating the observed CC_50_ values. For NHC-TP, we determined a selectivity of 22, similar to previous values of 11.5 to 13.2 and confirming that NHC-TP can be incorporated by h-mtRNAP (55). Selectivity for SOF-TP with h-mtRNAP could not be determined, consistent with SOF-TP being very poorly incorporated by h-mtRNAP (56, 57). By comparison, 4′-FlU-TP is used very efficiently by h-mtRNAP, with a selectivity of 4.1. Similarly, h-mtRNAP demonstrates efficient incorporation of 4′-FlA-TP, with a selectivity of 8.4. Efficient incorporation of 4′-FlA-TP by h-mtRNAP may indicate potential cytotoxic off-target effects, which could help interpret the observed CC_50_ values. However, NHC-TP similarly demonstrates efficient incorporation by h-mtRNAP. Indeed, while the incorporation of an analog by h-mtRNAP is an indicator of potential cytotoxicity, it is not the sole determinant, and the threshold for incorporation to elicit toxicity is unknown.

**Table 3:** Incorporation of nucleotide analogs by off-target polymerases

Replication strategy	Family	Enzyme	Compound	Selectivity^a^
Human DNA-dependent RNAP	Human	h-mtRNAP	RDV-TP	2,200;508^b^
			SOF-TP	>5000
			NHC-TP	22
			4′-FlU-TP	4.1
			4′-FlA-TP	8.4
Human DNA-dependent DNAP	Human	Pol γ	ddATP	0.66
			TFV-DP	210
			2′-deoxy-4′-FlA-TP	0.94
*E. coli* DNA-dependent DNAP	*E. coli*	Klenow -exo	ddATP	750
			TFV-DP	>5000
			2′-deoxy-4′-FlA-TP	4.8

h-mtRNAP, human mitochondrial RNA polymerase; Pol γ, human DNA polymerase gamma; Klenow -exo, DNA polymerase Klenow fragment (3′→5′ no exonuclease); RDV-TP, remdesivir triphosphate; SOF-TP, sofosbuvir triphosphate; NHC-TP, β-D-N4-hydroxycytidine triphosphate; 4′-FlU-TP, 4′-fluorouridine triphosphate; 4′-FlA-TP, 4′-fluoroadenosine triphosphate; ddATP, dideoxyadenosine; TFV-DP, tenofovir diphosphate.

a The selectivity of a polymerase for a nucleotide analog is calculated as the ratio of the *V*_max_/*K*_m_ values for the natural NTP and the analog, respectively.

b Data from Tchesnokov *et al*. 2019 (40).

Based on an existing cryo-EM structure of the h-mtRNAP transcription initiation complex (58), we generated a model of 4′-FlA-TP incorporation at the h-mtRNAP NTP-binding site (Fig. S13). The 4′-fluoro modification of 4′-FlA-TP fits well within the adjacent molecular surface of h-mtRNAP (Fig. S13*C*), supporting the efficient use of 4′-FlA-TP as a substrate. In contrast, the 1′-cyano substitution on RDV-TP clashes with h-mtRNAP residues (Fig. S13*D*), indicating poor incorporation with h-mtRNAP. This model aligns with previous modelling and the inefficient incorporation of RDV-TP (36).

Ribonucleoside analogs can be intracellularly converted to serve as substrates for host DNA polymerases. Ribonucleoside 5′-diphosphate (DP) is a metabolic intermediate in the activation pathway that can be used as a substrate by ribonucleotide reductase (RNR) (59). RNR is essential for the production of 2′-deoxyribonucleoside 5′-triphosphates used in DNA synthesis. Previous work demonstrated that NHC-DP could transit the RNR pathway, raising concerns about the potential mutagenic risk in humans (60). Therefore, we evaluated 2′-deoxy-4′-FlA-TP (2′-d-4′-FlA-TP) against the human DNA polymerase gamma (Pol γ) (Fig. S14), and *E.* *coli* DNA polymerase I (Klenow Fragment, KF) to further assess potential cytotoxicity (Table 3 and Table S5). KF was included as an additional measure of off-target evaluation. The active metabolite forms of dideoxyinosine and tenofovir (ddATP and TFV-DP, respectively) were included as benchmarks (Fig. S11) (61, 62, 63, 64). Against Pol γ, ddATP is incorporated very efficiently (selectivity of 0.66), whereas TFV-DP is incorporated 210-fold less efficiently than dATP. This outcome of substrate utilization by Pol γ is consistent with previous literature (50, 51). KF does not incorporate TFV-DP and selectively incorporates dATP 750-fold more efficiently than ddATP. Comparatively, we found Pol γ incorporates 2′-d-4′-FlA-TP with a selectivity of 0.94, and KF incorporates 2′-d-4′-FlA-TP 4.8-fold less efficiently than dATP. The ability of 4′-FlA-TP to be utilized as a substrate for RNR is unknown. Should 4′-FlA-TP be efficiently converted to 2′-d-4′-FlA-TP, there is potential for incorporation into host DNA. Ultimately, these findings suggest that, in addition to the efficient incorporation by viral polymerases, there are concerns regarding target specificity.

### Inhibition of host polymerases is not evident

**Figure 6:**
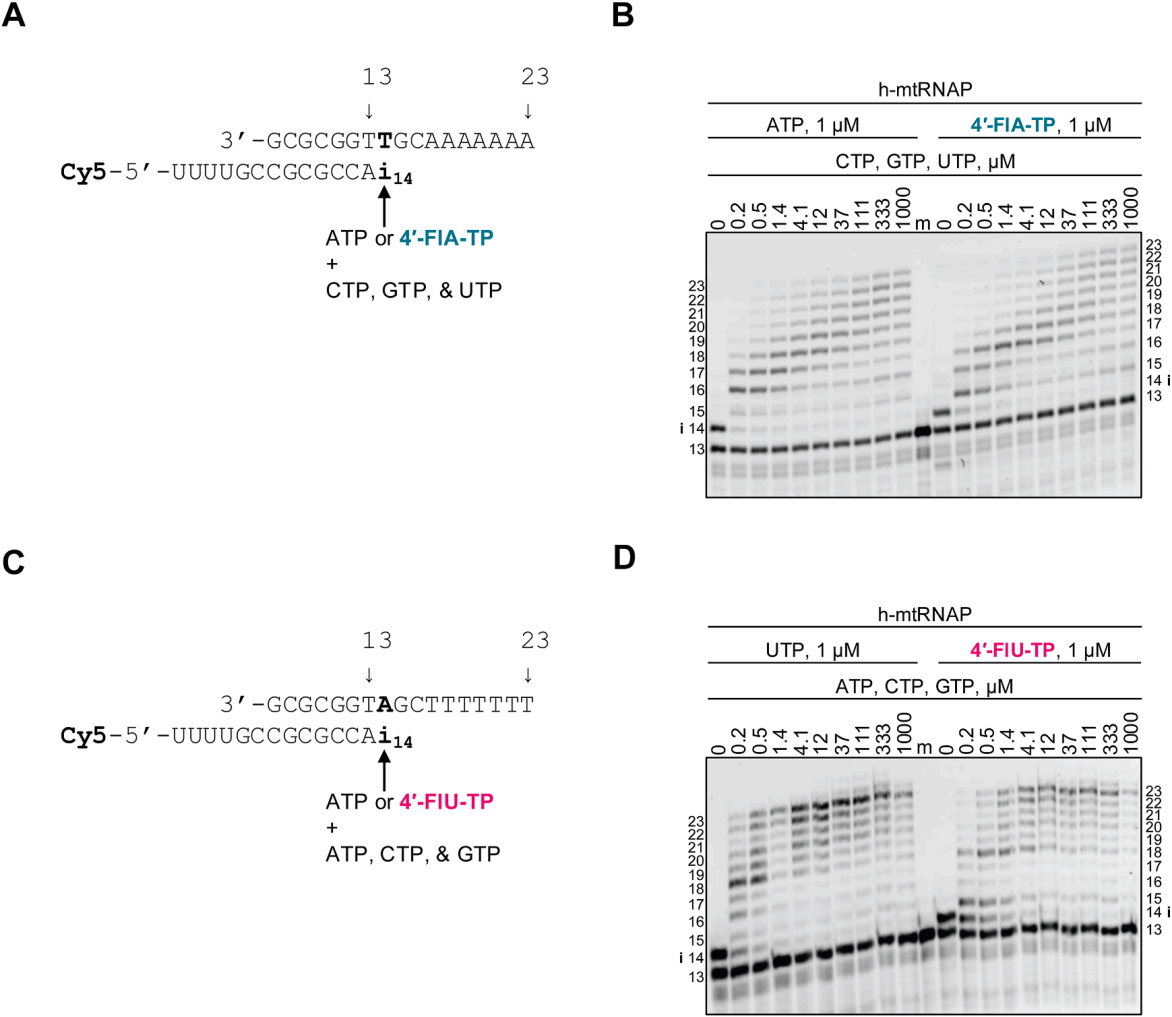
**h-mtRNAP DdRp-catalyzed RNA synthesis and inhibition patterns following a single incorporation of ATP or 4′-FlA-TP as a function of nucleotide concentration.***A,* RNA primer/DNA template supporting a single incorporation of ATP or 4′-FlA-TP at position 14 (“i”). The RNA primer is 5′-Cy5 labelled for signal detection of synthesized RNA products. *B,* migration pattern of RNA products resulting from h-mtRNAP DdRp-catalyzed RNA extension following incorporation of ATP or 4′-FlA-TP at position 14 (“i”) at increasing concentrations of CTP, GTP, and UTP. 5′-Cy5-labelled 13-nt primer serves as a size marker (m). *C,* RNA primer/DNA template supporting a single incorporation of UTP or 4′-FlU-TP at position 14 (“i”). The RNA primer is 5′-Cy5 labelled for signal detection of synthesized RNA products. *D,* migration pattern of RNA products resulting from h-mtRNAP DdRp-catalyzed RNA extension following incorporation of UTP or 4′-FlU-TP at position 14 (“i”) at increasing concentrations of ATP, CTP, and GTP. 5′-Cy5-labelled 13-nt primer serves as a size marker (m). h-mtRNAP, human mitochondrial RNA polymerase; 4′-FlA-TP, 4′-fluoroadenosine triphosphate; 4′-FlU-TP, 4′-fluorouridine triphosphate.

While efficient incorporation of 4′-FlA-TP and 2′-d-4′-FlA-TP by human and bacterial polymerases is indicative of off-target effects, cellular RNA synthesis may not necessarily be inhibited. We evaluated the patterns of RNA or DNA synthesis following incorporation of a single 4′-FlA-TP or 2′-d-4′-FlA-TP. For h-mtRNAP DdRp-catalyzed RNA synthesis, no discernible inhibitory effect in the primer-strand is observed following 4′-FlA-TP incorporation at position 14 (“i”) (Fig. 6, *A* and *B*). We also evaluated the potential inhibitory effect of subsequent nucleotide incorporation and found that incorporation of cytidine triphosphate (CTP) following 4′-FlA-TP incorporation is very subtly reduced by 1.8-fold relative to the natural scenario (Table S3). Similarly, no apparent inhibition is observed in the primer-strand following 4′-FlU-TP incorporation (Fig. 6, *C* and *D*). For Pol γ and KF, 2′-d-4′-FlA-TP shows no visible inhibition on primer-extension reactions (Fig. S15). In comparison, ddATP acts as an immediate chain terminator, disrupting DNA synthesis at the site of incorporation (Fig. S15*B*). Using the assays described above, inhibition of primer-extension reactions is not evident with 4′-fluorinated compounds.

## Discussion

**Figure 7:**
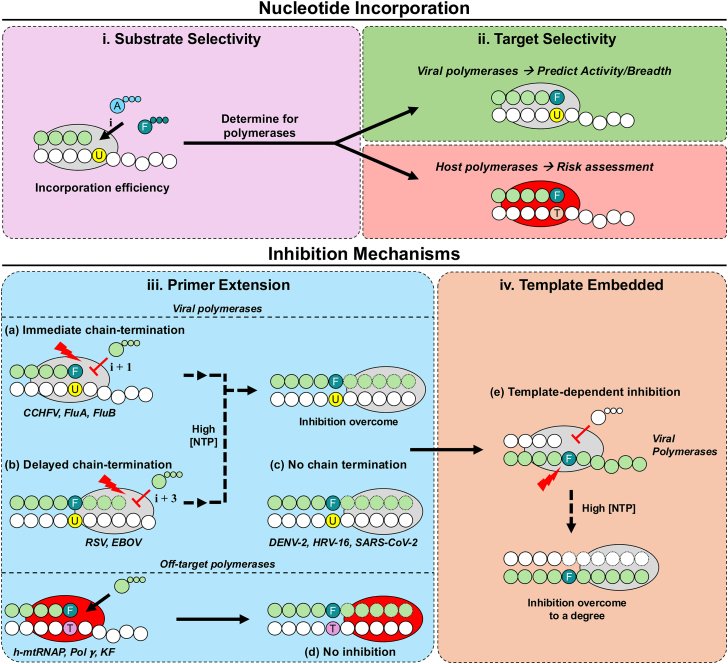
**Model of the mechanism of action of 4′-FlA.***i,* substrate selectivity - the investigation of 4′-FlA-TP incorporation efficiency relative to ATP. *ii,* target selectivity – 4′-FlA-TP is efficiently incorporated by all viral RdRp tested, indicative of potential broad activity. 4′-FlA-TP is also incorporated by nonviral polymerases, indicating potential off-target effects. *iii,* primer extension – once incorporated, 4′-FlA elicits heterogeneous effects against the viral RdRp, including *(a)* immediate chain-termination (Crimean-Congo hemorrhagic fever, FluB), *(b)* delayed chain-termination (RSV, EBOV), and *(c)* no chain-termination (DENV-2, HRV-16, SARS-CoV-2). When inhibition is observed, increased NTP concentrations appear to be sufficient to overcome inhibition. *d,* 4′-FlA shows no inhibitory effect against nonviral polymerases (h-mtRNAP, Pol γ, KF). *iv,* template embedded – 4′-FlA demonstrates a uniform mechanism against all viral RdRp by inhibiting NTP incorporation opposite the embedded 4′-FlA *(e)*. The priming strand is shown with *green* circles, *white* circles represent residues of the template, ATP is shown in *light blue*, 4′-FlA is shown in *teal*, uridine is shown in *yellow*, thymidine is shown in *pink*, the *red* lightning bolt indicates an inhibitory effect at a given position, the *light gray oval* represents a viral polymerase complex, and the *red* oval represents a nonviral polymerase complex. 4′-FlA, 4′-fluoroadenosine; 4′-FlA-TP, 4′-fluoroadenosine triphosphate; DENV-2, dengue virus 2; HRV-16, human rhinovirus 16; SARS-CoV-2, severe acute respiratory syndrome coronavirus 2; RSV, respiratory syncytial virus; EBOV, Ebola virus; CCHFV, Crimean-Congo hemorrhagic fever virus; FluA, influenza A virus; FluB, influenza B virus; NTP, nucleoside triphosphate.

4′-FlU shows a remarkable range of antiviral activity against diverse RNA viruses (25, 26, 27, 28, 29, 30, 31, 32, 33). Depending on the pathogen, the *in vitro* potency is often non-inferior or even superior to approved NAs with a broad-spectrum of targets (10, 11, 12, 13, 14, 15, 16, 17, 18, 19, 20, 21, 22, 23). These findings warrant further investigation into this class of compounds. Here, we studied the spectrum of antiviral activity of the related 4′-FlA and utilized a comprehensive platform to determine biochemical attributes of the active 4′-FlA-TP form (Fig. 7). 4′-FlA demonstrates potent antiviral activity against each of the following viruses: DENV-2, SARS-CoV-2, HRV-16, RSV, EBOV, CCHFV, FluA, and FluB. Our data also show efficient rates of incorporation of 4′-FlA-TP by the corresponding polymerase complexes. Substrate selectivity measurements suggest that the NA can efficiently compete with natural ATP pools. However, efficient incorporation is also seen by host enzymes, which can be indicative of off-target effects (49, 50, 51, 52). Both 4′-FlA-TP and 4′-FlU-TP are incorporated by h-mtRNAP, and the deoxy-NTP counterpart of 4′-FlA-TP, 2′-d-4′-FlA-TP, is efficiently incorporated by nonviral DNA polymerases. Together, these data raise concerns regarding target specificity. However, inhibition of host enzymes is not evident, and a favorable SI was observed in some cases, such as CCHFV.

The pattern of inhibition within primer-extension reactions appears to depend on the nature of the viral polymerase, which is consistent with reports on 4′-FlU-TP (29, 30). The mechanism of action of numerous other 4′-modified NAs has been studied, and chain-termination is commonly reported as the dominant mechanism of inhibition (29, 36, 49, 65, 66, 67). Examples include 4′-azidocytidine (balapiravir) and 2′-deoxy-2′-β-fluoro-4′-azidocytidine that were originally developed for hepatitis C virus (65, 68, 69, 70, 71). Lumicitabine, a 2′-fluoro-4′-chloromethyl-cytidine analog, was developed for RSV infection (66, 72). GS-7682, an investigational 4′-cyano-modified adenosine analog, is effective against RSV and HRV (73). 4′-ethynyl-2-fluoro-2′-deoxyadenosine, developed for the treatment of HIV-1 infection, elicits chain-termination by impairing enzyme translocation (67, 74). 4′-FlU-TP shows different effects on primer extension reactions, depending on the nature of the viral polymerase (29, 30). These heterogeneous patterns of inhibition are also seen with 4′-FlA-TP; however, template-dependent inhibition by 4′-FlA provides a unifying mechanism across the viral RdRps included in this study. When embedded in the templating-strand, 4′-FlA-MP inhibits the incorporation of the complementary UTP. Template-dependent inhibition has also been reported for RDV (12, 36, 37, 41). This mechanism needs to be considered for compounds that contain the 3′-hydroxyl group, especially if chain-termination is not absolute. The observation that both 1′-cyano and 4′-fluoro modifications can mediate broad-spectrum antiviral activity through the same mechanism could also be exploited in drug development efforts. The structural differences between these compounds are more sensitive to rates of incorporation, which likely diminishes the antiviral effects of RDV against several segmented negative-sense RNA viruses.

Structural studies are necessary to deepen our understanding of how the 4′-fluoro modification affects substrate use. Our cryo-EM structure provides guidance in this regard and offers insight into the incorporation and subsequent effects of 4′-FlA-TP in the primer strand. It is now of particular interest to determine the structural requirements for template-dependent inhibition. Our structural modelling suggests that this effect may be due to a higher likelihood of steric clashes between the 4′-fluoro modification in the template strand and surrounding residues. However, it is important to note that nucleotide incorporation may accompany conformational changes that our static modelling cannot capture, further highlighting the need for structural studies. It is not evident that such a mechanism may also affect host polymerases. The nature, quantity, and biological relevance of cellular RNA templates with incorporated NAs are complex factors that remain to be studied. Modified RNAs generated by host polymerases have numerous functions that may require consideration to assess potential toxicities (75).

In conclusion, the 4′-fluoro modification provides a promising scaffold with a broad-spectrum of antiviral activity that can be ascribed to both efficient incorporation and a unifying template-dependent mechanism of inhibition. However, substrate utilization by host polymerases raises concerns. 4′-FlU and 4′-FlA provide viable starting points for the design of derivatives possessing additional modifications to improve selectivity for viral polymerase targets. The biochemical approach described in this study may guide and help to de-risk such efforts (Fig. 7). Efficacy and safety studies should then accompany the identification of appropriate medical settings of potential therapeutic benefit. Treatment of infections by high-consequence pathogens should be considered in this context.

## Experimental procedures

### Chemicals

NTPs were purchased from GE Healthcare. [α-^32^P]-GTP was purchased from PerkinElmer (Revvity). NHC-TP and baloxavir acid were purchased from MedChemExpress. ddATP was purchased from Roche (Mannheim, Germany). kCMN996 (RDV, GS-441524) was purchased from Combi Blocks. TFV-DP was purchased from Toronto Research Chemicals. mCOT466 (4′-FlA), mCOU991 (4′-FlA-TP), mCQT310 (2′-d-4′-FlA-TP), and kCOT700 (4′-FlU-TP) were synthesized as follows: mCOT466 (4′-FlA): WO2024227159 – 4′-substituted nucleosides and nucleotides as antiviral agents – Google Patents (76); mCOU991 (4′-FlA-TP): See “Synthetic chemistry” in supporting information (Fig. S16); mCQT310 (2′-d-4′-FlA-TP): See “Synthetic chemistry” in supporting information (Fig. S17); kCOT700 (4′-FlU-TP): Synthesized according to a previously published protocol (77).

### DENV-2, HRV-16, SARS-CoV-2, RSV, and FluB cell-based assays

The preparation of dengue virus (Dengue virus type 2 or DENV-2 16,681 strain), human rhinovirus (HRV-16), SARS-CoV-2 (USA/WA1/2020), and RSV-A (A2 strain) viral stocks and subsequent assessment and analysis of the antiviral activity and cytotoxicity of 4′-FlA (or mCOT466) against these viruses in various cell lines have been previously described in Google Patent WO2024227159, “4′-substituted nucleosides and nucleotides as antiviral agents” (76). The assay for evaluating the efficacy of 4′-FlA against FluB in Madin-Darby canine kidney cells has been previously described (78). Cell lines were validated upon receipt (per ATCC QC specifications) and by assay performance and viral susceptibility. Cells were regularly tested for *mycoplasma* using the MycoAlert Detection Kit (Lonza, LT07–318) to confirm the experiments were free from contamination.

### EBOV antiviral assays

HeLa cells (ATCC CCL-2) were seeded at 4000 cells/well 24h prior to infection. Experimental compounds were prepared as 5 mM stocks in Dimethyl sulfoxide (DMSO, Sigma-Aldrich) and further diluted to 200 μM. Cell culture media was aspirated and replaced with 40 μl of DMEM (Dulbecco’s Modified Eagle Medium, Gibco) containing 2% FBS (Fetal Bovine Serum, GeminiBio) and 100 units/ml of P/S (Penicillin-Streptomycin, Gibco). Compounds were dispensed onto the cell-containing plates using an iDOT liquid handler (Dispendix) in a 10-point dose-response curve in triplicate, ranging from 20 μM to 0.04 μM. All wells were backfilled to a final volume of 0.2 μl DMSO. Outer wells were excluded from compound addition due to edge effects. Following compound treatment, cells were challenged with Ebola Mayinga virus (EBOV; isolate Ebola virus/H.sapiens-tc/COD/1976/Yambuku-Mayinga) within the BSL-4 laboratory. Virus inoculum was prepared in DMEM supplemented with 2% FBS and 1xPenicillin/Streptomycin solution to yield a final multiplicity of infection of 0.2, and 10 μl was added per well. The plates were then incubated for 40 h at 37 °C before being inactivated in 10% formalin. Following inactivation by formalin, plates were removed from the BSL-4 laboratory and washed in 1x phosphate-buffered saline (PBS, Thermo Fisher Scientific) permeabilized with 0.1% (v/v) Triton X (Acros Organics) for 15 min and blocked with 3.5% bovine serum albumin (BSA, 1 h). Cells were stained with mouse anti-EBOV glycoprotein (4F3, IBT Bioservices) for 3 h at room temperature. Following a wash with 1xPBS, the secondary antibody, donkey anti-mouse AlexaFluor 488 (Invitrogen), was added and incubated overnight at 4 °C. Plates were then washed and counterstained with Hoechst 33342 (Thermo Fisher Scientific). Plates were imaged using a Cytation 1 automated microscope (BioTek) using the blue and green fluorescence channels. Image analysis was performed using CellProfiler (Broad Institute, v4.2.6), where pipelines were configured to quantify total EBOV-infected cells and nuclei. Infection rates were calculated for each well and normalized to the DMSO control wells. Cell viability was assessed per well, with values normalized to the DMSO controls. Dose-response curves were generated using GraphPad Prism (v10.2.3) and the four-parameter logistic model ([agonist] vs response, variable slope). 4′-FlA was also screened against the EBOV minigenome assay using HEK293T (ATCC CRL-3216) cells as previously described (79). Cells were validated by assay performance only and were intermittently evaluated for *mycoplasma* contamination.

### Rescue of recombinant CCHFV and subsequent dose-response inhibition

Recombinant Crimean-Congo hemorrhagic fever virus expressing the Zs-Green fluorescent protein (rCCHFV-ZsGreen) was rescued as previously described (80, 81). Briefly, baby hamster kidney cells (BHK-21; ATCC CCL-10) were seeded in 6-well plates (1 × 10^6^ cells per well) 1 day prior to transfection. Culture medium was removed and replaced with Opti-MEM for plasmid transfection. Cells were transfected with a pT7 plasmids encoding the CCHFV IbAr10200 gene segments S, M, and L (1 μg, 2.5 μg, and 1 μg, respectively), along with pCAGGS helper plasmids encoding CCHFV nucleoprotein (NP) and L proteins, and a codon-optimized bacteriophage T7 RNA polymerase (0.66 μg, 0.33 μg, and 1 μg, respectively), using Lipofectamine 3000 (Thermo Fisher Scientific) according to the manufacturer’s instructions. The S genome segment contains Zs-Green inserted upstream of the NP gene separated by a porcine teschovirus-1 2A peptide linker sequence (P2A). Plasmids were provided by Éric Bergeron and César G. Albariño (Centers for Disease Control and Prevention, CDC). At 24 h post-transfection (hpt), Opti-MEM was replaced with cell culture medium. Cell culture supernatants were collected at 96 hpt and used to infect African green monkey kidney epithelial cells (Vero E6; ATCC CRL-1586) in T75 flasks for virus amplification and generation of viral stocks.

Vero E6 cells were seeded in 96-well clear-bottom black plates (2 x 10^4^ cells/well) in quadruplicate 1 day before infection. Cells were infected with rCCHFV Zs-Green at a MOI of 0.005. Following 1 h of viral adsorption, media containing 2-fold serial dilutions of inhibitors (starting at 50 μM), were added directly to the viral inoculum. At 72 h post-infection, cells were inactivated with 10% formalin and stained with DAPI. Fluorescence intensities of Zs-Green and DAPI were quantified using a GloMax plate reader (Promega) and Zs-Green signal was normalized to mock-treated, infected cells, whereas DAPI signal was normalized to mock-treated, mock-infected cells for EC_50_ and CC_50_ determinations, respectively. All work involving rCCHFV were performed in BSL-4 facilities at the Texas Biomedical Research Institute).

### FluA/A549 high-content imaging assay and cytotoxicity counter screen

FluA infection was quantified by immunofluorescence-based high-content imaging multi-cycle assay as previously described for influenza antiviral screening assays, with minor modifications (82). Compounds were acoustically transferred using a Labcyte Echo liquid handler into collagen I-coated 384-well μclear-bottom plates (Greiner, 781090-2B). A549 (ATCC CCL-185) cells were mixed with Influenza A virus A/WSN/1933 (H1N1) (WSN/33) at a MOI of 0.01 and seeded at 4.0 × 10^3^ cells per well in 30 μl F-12K medium supplemented with 1% BSA (Sigma), and 0.5 μg/ml TPCK-treated irradiated trypsin (Worthington). Plates were incubated for 48 h at 37 °C with 5% CO_2_. Cells were fixed with 4% formaldehyde for 15 min at room temperature, washed with PBS containing 0.05% Tween-20 (PBS-T), permeabilized with 0.25% Triton X-100 for 10 min, and blocked with SuperBlock T20 (Thermo Fisher Scientific) for 1 h. Plates were incubated overnight at 4 °C with goat anti-influenza A polyclonal antibody (United States Biological, I7650-05E-ML550; 1:1000 dilution) and 8 μM DAPI in blocking buffer. After washing with PBS-T, plates were sealed and imaged using an ImageXpress Micro Confocal High-Content Imaging System (Molecular Devices) with a 10x objective (four fields per well). Images were analyzed using MetaXpress (v6.7.2.290). DAPI staining was used to identify total nuclei, and influenza A immunofluorescence was used to quantify infected cells. For cytotoxicity assessment, A549 (ATCC CCL-185) cells were maintained at 37 °C with 5% CO_2_ in humidified incubators and cultured in F-12K medium supplemented with 10% heat-inactivated FBS, 100 IU/ml penicillin, and 100 μg/ml streptomycin. 300 A549 cells per well in assay media (same as maintenance media but with 2% FBS) were seeded in 1536-well white tissue culture-treated plates (Corning 9006BC) containing compounds transferred acoustically in three-fold serial dilutions starting at 80 μM. Cells were incubated for 48 h, after which viability was quantified using CellTiter-Glo (Promega G7573; 2 μl of 50% reagent diluted in water) and luminescence measured on a CLARIOstar plate reader (BMG Labtech). High-content image data and cytotoxicity data were processed using Genedata Screener (v21.0.3). IAV/A549 assay data were normalized to DMSO controls and inhibitor controls (0.2 μM baloxavir for antiviral activity and 10 μM puromycin for infected-cell toxicity). For cytotoxicity assays, 80 μM puromycin served as the positive control. Dose-response curves were generated from technical triplicates using a four-parameter Hill equation. Cell lines were validated upon receipt (per ATCC QC specifications) and by assay performance and viral susceptibility. Cells were regularly tested for *mycoplasma* using the MycoAlert Detection Kit (Lonza, LT07-318) to confirm the experiments were free from contamination.

### Nucleic acids

The following synthetic oligos were used in this study. The portion of the template that is underlined is complementary to the primer used. Oligos requiring radiolabeling were 5′-monophosphorylated. For assays with most viral RdRps, the RNA template 3′-UGCGCUAGAAAAAAp-5′ was used for analog selectivity, pattern of inhibition, and UTP incorporation at position “i + 1” experiments. Analog selectivity, pattern of inhibition, and UTP incorporation at position “i + 1” experiments for EBOV RdRp were evaluated using 3′-UGCGCUAGUUUAUUp-5′. Pattern of inhibition for RSV RdRp was also evaluated using 3′-UGCGCUAGUUUAUUp-5′. 3′-UGCGCUUUUUXUUGUUGUUUp-5′ was used for the evaluation of nucleotide incorporation opposite a template-embedded analog in comparison to the natural scenario, where X represents either templated adenosine (Template “A”) or templated 4′-FlA (Template “F”). These RNA templates were produced as previously described (41). For h-mtRNAP, the DNA template 3′-GCGCGGTTGCAAAAAAA-5′ and the RNA primer 5′-Cy5-UUUUGCCGCGCCA-3′ were used for 4′-FlA-TP selectivity, pattern of inhibition, and CTP incorporation at position “i + 1” experiments. 3′-GCGCGGTGACTATTATTA-5′ DNA template and 5′-FAM-UUUUGCCGCGCCA-3′ RNA primer were used for NHC-TP selectivity. 3′-GCGCGGTAGCTTTTTTT-5′ DNA template and 5′-Cy5-UUUUGCCGCGCCA-3′ RNA primer were used for 4′-FlU-TP selectivity. For DNA Pol γ and KF, 3′-TAAGACTGATTTTCCCAGACTCCCTATCAGAGAACG-5′ DNA template and 5′-Cy5-ACTAAAAGGGTCTGAGGGAT-3′ DNA primer, based on Baldwin *et al.* (83), were used for analog selectivity and pattern of inhibition experiments. 5′-monophosphorylated RNA primers and templates were purchased from Dharmacon. 5′-triphosphorylated RNAs were purchased from ChemGenes, then capped and radiolabeled using [α-^32^P]-GTP, the mRNA cap 2′ O-methyltransferase, and vaccinia capping system from New England BioLabs. 5′-fluorescently labelled (Cy5) primers and their complementary templates were purchased from IDT.

### Expression and purification of enzymes

The design rationale, expression, and purification of DENV-2, HRV-16, SARS-CoV-2, RSV, EBOV, CCHFV, FluB, h-mtRNAP, and Pol γ used in this study have been previously described (12, 36, 39, 40, 41, 46, 65, 84). DNA polymerase Klenow fragment (3′→5′ no exonuclease) was purchased from New England Biolabs (M0212S, Ipswich). The baculovirus expression system was also employed for the FluA H3N2 PA/PB1/PB2 complex. The pFastBac-1 (Invitrogen) plasmid with codon-optimized synthetic DNA sequences (GenScript) encoding for FluA PA (PA_AAA43597.1), PB1 (PB1_AAA43579.1), and PB2 (PB2_AAA43613.1) was designed and used as starting material for protein expression, based on previously reported by others (85, 86, 87). We utilized the MultiBac (Geneva Biotech) system for protein expression in Sf9 insect cells (Invitrogen) according to the published protocols (88, 89). The FluA RdRp components (PA, PB1, PB2) were expressed as individual recombinant proteins controlled by individual polyhedrin promoters. The resulting FluA RdRp complex is comprised of the PA subunit, the PB1 subunit, and the PB2 subunit with a C-terminal 8xHis tag and Protein A tag. Sf9 cell pellets expressing FluA RdRp complex were lysed, and the polymerase was purified as previously reported (37, 84). Protein purity and identity were confirmed by SDS-PAGE and mass spectrometric analysis (Dr Jack Moore, Alberta Proteomics and Mass Spectrometry Facility, University of Alberta).

### *In vitro* gel-based assays

RNA synthesis assays, data acquisition, and quantification were performed as we previously described (36, 37, 39, 40, 65). Reactions containing viral RdRp and non-5′-labeled primer were provided with 0.1 μM [α-^32^P]-GTP for visualization of RNA synthesis. For experiments determining kinetic parameters, enzyme concentrations and the time point for each reaction were optimized to ensure incorporation of the first nucleotide was within the linear range of product formation. Standard reaction mixtures contained purified polymerase complex, 25 mM Tris-HCl pH 8.0, 200 μM primer, 2 μM template (10 μM for CCHFV in analog selectivity, pattern of inhibition, and “i + 1” experiments), 0.1 μM [α-^32^P]-GTP, 0.5 mM EDTA (0.25 mM for HRV-16, SARS-CoV-2, EBOV, Pol γ, and KF), and various nucleotide concentrations. For EBOV, glycerol was added to reaction mixtures to equal 5%. For h-mtRNAP evaluation of 4′-FlA-TP and 4′-FlU-TP, 50 nM of primer and 100 nM of template were provided, while NHC-TP investigation utilized primer-template strands which were annealed before their addition into the mixture (50 nM) as previously described (65). For Pol γ and KF, 50 nM of pre-annealed primer-template was supplemented. Reactions with SARS-CoV-2, h-mtRNAP, Pol γ, and KF also contained 50 mM NaCl. Mixtures were incubated for 5 to 10 min at 23 °C (h-mtRNAP), 30 °C (viral RdRps), or 37 °C (Pol γ and KF), and RNA synthesis was initiated by the addition of 2.5 mM (HRV-16, SARS-CoV-2, EBOV, h-mtRNAP, and Pol γ) or 5 mM (DENV-2, RSV, CCHFV, FluB, and KF) MgCl_2_ and incubated. The reaction duration varied among enzymes: DENV-2, HRV-16, RSV, and FluB reactions were stopped after 10 min; SARS-CoV-2 and Pol γ reactions were stopped after 2 min; KF reactions were stopped after 1 min; CCHFV reactions were stopped after 15 min; EBOV reactions were stopped after 20 min; and h-mtRNAP reactions with 4′-FlA-TP and NHC-TP were stopped after 4 min, whereas reactions with 4′-FlU-TP were stopped after 8 min. The capped primer extension assay utilized purified FluA or FluB RdRp enzyme, 25 mM Tris-HCl pH 8.0, 50 mM NaCl, 5 mM MgCl_2_, 0.5 mM EDTA, 10 μM baloxavir acid, 1 μM vRNA (equimolar 5′ (5′ pAGUAGUAACAAGAG 3′) and 3′ (5′ UAUACCUCUGCUUCUGCU 3′) RNAs) incubated for 5 min at 30 °C. Reactions were started with the addition of 0.1 μM cap1 radiolabeled primer (5′m^7^G-AmAUCUAUAAUAGC; synthesized according to manufacturer protocols and purified as described previously) and nucleotides at various concentrations, then allowed to proceed for 10 min at 30 °C (90). All reactions were stopped by the addition of an equal volume of a formamide/EDTA (50 mM) mixture. Reaction products were then incubated at 95 °C for 5 min and resolved by 20% PAGE containing 8 M urea, 89 mM Tris base, 89 mM Boric acid, and 2 mM EDTA, using 1X TBE running buffer. Radiolabeled or fluorescent signals were then visualized using an Amersham Typhoon biomolecular imager (Cytiva)and quantified in Cytiva ImageQuant TL software (https://www.cytivalifesciences.com/en/us/products/items/imagequant-tl-analysis-software-p-28619?psmenu=2). The data were analyzed using GraphPad Prism 10 (GraphPad Software, Inc, https://www.graphpad.com). To calculate selectivity, the fraction of products generated at position “i” out of the total signal in the lane was determined and plotted against the concentration of the nucleotide or NA. The kinetic parameter *V*_max_ was defined as the product fraction of extended primer relative to the total signal observed in each lane. *K*_m_ reflects the nucleotide concentration at half the *V*_max_ value. *V*_max_/*K*_m_ determines the efficiency of single nucleotide incorporation for a given nucleotide. To compare the efficiency of nucleotide incorporation, a selectivity value is determined as the ratio of the incorporation efficiency of the natural nucleotide to the incorporation efficiency of the analog. Incorporation efficiency of the subsequent nucleotide following either NTP or analog incorporation (at position “i + 1”) was calculated as the fraction of RNA synthesized products generated at position “i + 1” and beyond, divided by the total signal in the lane, and plotted against the concentration of NTP incorporated at “i + 1”. Inhibition (fold) compares the efficiency of NTP incorporation following 4′-FlA-TP incorporation to the efficiency of NTP incorporation following ATP incorporation.

### Cryo-EM sample preparation

For cryo-EM of SARS-CoV-2 RdRp complex with RNA and 4′-FlA-TP, the nsp12, nsp7, and nsp8 subunits were expressed separately. The nsp12 gene with a C-terminal TEV cleavage site and a Twin-Strep-Tag was codon-optimized and inserted into the pEZT-BM vector. HEK293F cells were transfected with 1 mg DNA and 3 mg PEI per liter of cells. After 5 days, cells were harvested, and pellets were resuspended in lysis buffer containing 25 mM Tris, pH 7.4, 300 mM NaCl, 1 mM MgCl_2_, 0.1% IGEPAL CA-630, 1 mM DTT, 10% glycerol, and protease inhibitor cocktail. Following cell lysis by sonication, the lysate was clarified by centrifugation at 30,000×*g* for 30 min. The supernatant was applied to Strep-Tactin XT resin (IBA Lifesciences) for affinity purification. After extensive washing, the bound protein was eluted overnight with Buffer BXT (IBA Lifesciences) and further purified by size exclusion chromatography on a Superdex 200 10/300 column (Cytiva) equilibrated with buffer containing 25 mM Tris pH 7.4, 300 mM NaCl, 0.1 mM MgCl_2_, and 1 mM DTT. The nsp7 and nsp8 subunits were expressed in BL21(DE3) *Escherichia coli* cells. Protein expression was induced with 1 mM IPTG at an OD_600_ of 0.7, and cultures were incubated overnight at 18 °C. Harvested cells were lysed by sonication in a buffer containing 10 mM Tris, pH 7.4, 300 mM NaCl, 30 mM imidazole, and 5 mM β-mercaptoethanol. His-tagged proteins were purified using Ni-NTA affinity chromatography followed by TEV protease cleavage and dialysis to remove the His-tag. For RdRp complex assembly, purified nsp12 was incubated overnight with nsp7 and nsp8 at a molar ratio of 1:5:5. The assembled complex was further purified by size exclusion chromatography and concentrated in a final buffer containing 25 mM Tris, pH 7.4, 100 mM NaCl, 2 mM MgCl_2_, and 1 mM DTT. For cryo-EM sample preparation, the RdRp complex was incubated with dsRNA substrate (Integrated DNA Technologies; template strand: 5′-UUUUUUUUUUAUAACUUAAUCUCACAUAGC-3′; primer strand: 5′-GCUAUGUGAGAUUAAGUUAU-3′) as previously described by Yin *et al.* (42), and 4′-FlA-TP at a molar ratio of 1:2:10 for 2 h at 4 °C before grid preparation. Grid preparation was performed by mixing 3 μl of RdRp complex containing 4′-FlA-TP and dsRNA at 3 mg/ml with 0.5 μl of n-dodecyl β-D-maltoside to achieve a final concentration of 0.00128%. The sample was immediately applied to glow-discharged 1.2/1.3300-mesh UltraAuFoil grids and plunge-frozen using a Vitrobot Mark IV (Thermo Fisher Scientific) under controlled conditions at 4 °C chamber temperature, 100% humidity, 10 s wait time, and 3 s blotting time before plunging into liquid ethane.

### Cryo-EM data collection and image processing

Cryo-EM data acquisition was performed on a 200 kV Glacios (Thermo Fisher Scientific) equipped with a Falcon 4i direct electron detector. Automated image collection was conducted using EPU software (Thermo Fisher Scientific, https://www.thermofisher.com/us/en/home/electron-microscopy/products/software-em-3d-vis/epu-software.html) at a nominal magnification of 190,000× with a pixel size of 0.718 Å. A total of 7824 movies were collected and processed using CryoSPARC (91). Motion correction with dose weighting and CTF estimation were performed using CryoSPARC Live. For image processing, particles were initially picked using blob picker followed by 2D classification. Template-based particle picking was then performed using selected 2D class averages as templates. Multiple rounds of 2D classification were conducted to obtain clean particle datasets. Selected particles were subjected to *ab initio* reconstruction followed by heterogeneous refinement to separate different conformational states. The 3D model corresponding to the RdRp complex with RNA was selected from heterogeneous refinement and subjected to non-uniform refinement. An additional 3D classification was performed to further clean the particle set. To select the RdRp complex containing the 4′-FlA molecule, a mask was applied to the active site, and 3D variability analysis was performed, resulting in 48,482 particles used for the final map reconstruction. Finally, local refinement was conducted with a mask applied to the core region of RdRp after removing flexible domains, yielding a final structure at 3.38 Å resolution.

### Model building and refinement

The atomic model was initially built using PDB: 7BV2 as a starting template (42). Model refinement was carried out through Rosetta relaxed refinement protocols (92), followed by iterative rounds of manual adjustment in Coot and real-space refinement using Phenix (93, 94). Model quality was monitored throughout the refinement process by calculating EMRinger and MolProbity scores after each refinement cycle (95, 96). Structural visualization and figure preparation were performed using ChimeraX and PyMOL software (97, 98). Comprehensive model statistics and validation data are presented in Table S4 and Fig. S10, *C* and *D*.

### h-mtRNAP modelling studies

Modelling of 4′-FlA-TP and RDV-TP within the NTP-binding site of h-mtRNAP was performed using a previously determined cryo-EM structure (PDB: 9R96) (58). The incoming GTP was replaced with either 4′-FlA-TP and RDV-TP, and modelled manually using Coot and Phenix, for the analogs to maintain base-pairing and chelation with the catalytic Mg^2+^ ions at the enzyme active site (93, 94). Structural visualization and figure preparation were performed using PyMOL software (98).

## Data availability

The atomic model and cryo-EM density map generated in this study have been deposited in the PDB (accession number: 12SN) and EMDB (accession number: EMD-76733), respectively. The data underlying this article are available in the article itself, its supplementary material, and the Open Science Framework online repository at https://doi.org/10.17605/OSF.IO/A5R4D.

## Supporting information

This article contains supporting information (40, 42, 58).

## Conflict of interest

The authors declare that they have no conflicts of interest with the contents of this article.
