# Supplementary material for: Substrate and target selectivity of 4′-fluoroadenosine against viral and host polymerases
Source: J Biol Chem. 2026 Jul 6;302(8):113316. doi: 10.1016/j.jbc.2026.113316 (PMC13487286; doi:10.1016/j.jbc.2026.113316)
Supplement: Supplementary file 1 [file mmc1.docx]

Supporting Information

**Substrate and target selectivity of 4′-fluoroadenosine against**

**viral and host polymerases**

Simon M. Walker^1^, Arlo J. Loutan^1^, Egor P. Tchesnokov^1^, Dana Kocincova^1^, Calvin J. Gordon^1,2^, Ruby A. Escobedo^4^, Nathaniel Jackson^4^, Olivia A. Vogel^5^, Kim Morsheimer^6^, Suncheol Park^7^, Anant Gharpure^7^, Ixchel Urbano^8^, Mina Heacock^8^, Zhong Chen^8^, Khushboo Pathak^8^, Karen C. Wolff^8^, Lauren Huerta^8^, Malina A. Bakowski^8^, Laura Riva^8^, Anil K. Gupta^8^, Chenguang Yu^8^, Kalyan Das^1,2,3^, Luis Martinez-Sobrido^4^, Christopher F. Basler^5^, Robert Davey^6^, Ian A. Wilson^7^, Andrew B. Ward^7^, Sumit Chanda^8^, Arnab K. Chatterjee^8^, Matthias Götte^1*^

^1^ Department of Medical Microbiology and Immunology, University of Alberta, Edmonton, AB, Canada

^2^ Li Ka Shing Institute of Virology, University of Alberta, Edmonton, AB, Canada

^3^ Molecular Structural and Translational Virology, Department of Microbiology, Immunology and Transplantation, Rega Institute for Medical Research, KU Leuven, Leuven, Belgium

^4^ Texas Biomedical Research Institute, San Antonio, TX, USA

^5^ Department of Microbiology, Icahn School of Medicine at Mount Sinai, New York, NY, USA

^6^ Department of Virology, Microbiology and Immunology, Boston University, Boston, MA, USA

^7^ Department of Integrative Structural and Computational Biology, The Scripps Research Institute, La Jolla, CA, USA

^8^ Calibr-Skaggs Institute for Innovative Medicines, The Scripps Research Institute, La Jolla, CA, USA

**This file includes:**

- Synthetic chemistry
- Tables S1 to S5
- Figures S1 to S17

**Synthetic chemistry**: 2′-deoxy-4′-FlA-TP (mCQT310 or V2870202)

**Synthetic chemistry**: 4′-FlA-TP (mCOU991 or V2583902)

**Table S1:** Selectivity values for 4′-FlA-TP against viral RdRp.

**Table S2**: Selectivity values for RDV-TP and 4′-FlA-TP against influenza RdRp using 13-nt capped primer system.

**Table S3**: Inhibitory effect of the incorporated analog-monophosphate on the incorporation of the subsequent nucleotide.

**Table S4**: Cryo-EM data collection, refinement, and validation statistics for SARS-CoV-2 RdRp complex with dsRNA and 4′-FlA-TP.

**Table S5**: Selectivity values for nucleotide analogs against off-target polymerases.

**Figure S1.** Selective incorporation of 4′-FlA-TP by RSV RdRp.

**Figure S2.** Selective incorporation of 4′-FlA-TP by FluA RdRp in the capped primer extension assay.

**Figure S3.** HRV-16 and SARS-CoV-2 RdRp-catalyzed RNA synthesis and inhibition patterns following a single incorporation of ATP or 4′-FlA-TP as a function of nucleotide concentration.

**Figure S4.** CCHFV RdRp-catalyzed RNA synthesis and inhibition patterns following a single incorporation of ATP or 4′-FlA-TP as a function of nucleotide concentration.

**Figure S5.** RSV RdRp-catalyzed RNA synthesis and inhibition patterns following a single or multiple incorporations of ATP or 4′-FlA-TP as a function of nucleotide concentration.

**Figure S6.** FluA and FluB RdRp-catalyzed RNA synthesis and inhibition patterns following multiple incorporations of ATP or 4′-FlA-TP as a function of nucleotide concentration.

**Figure S7**. Inhibitory effect of RNA extension by CCHFV RdRp following 4′-FlA-TP incorporation.

**Figure S8.** Template-dependent inhibition of HRV-16, SARS-CoV-2, EBOV, and CCHFV RdRp by a single embedded 4′-FlA residue at position 11.

**Figure S9.** Impairment of NTP incorporation opposite a template-embedded 4′-FlA.

**Figure S10.** Structural basis for 4′-FlA incorporation by the SARS-CoV-2 RdRp and cryo-EM data quality.

**Figure S11.** Chemical structures of additional nucleoside and nucleotide analogs investigated in this study.

**Figure S12.** Incorporation of nucleotide analogs by h-mtRNAP.

**Figure S13.** Modelled binding of 4′-FlA-TP in the human mitochondrial RNA polymerase (h-mtRNAP) initiation catalytic complex.

**Figure S14.** Incorporation of nucleotide analogs by Pol γ.

**Figure S15.** Pol γ and Klenow DdDp-catalyzed DNA synthesis and inhibition patterns following a single incorporation of dATP or 2′-d-4′-FlA-TP as a function of nucleotide concentration.

**Figure S16.** Chemical synthesis scheme of 4′-fluoroadenosine triphosphate (4′-FlA-TP).

**Figure S17.** Chemical synthesis scheme of 2′-deoxy-4′-fluoroadenosine triphosphate (2′-d-4′-FlA-TP).

**Synthetic chemistry**

4′-FlA-TP (mCOU991 or V2583902) (Scheme in Figure S16)

To a solution of Compound **1** (130 mg, 457 μmol, 1.00 *eq*), proton sponge (97.8 mg, 457 μmol, 1.00 *eq*) in PO (OMe)_3_ (1.30 mL) was added POCl_3_ (104 mg, 685 μmol, 63.5 μL, 1.80 *eq*) at -10 °C. The mixture was stirred at 25 °C for 1 hr under N_2_. **LCMS** (RT = 0.280min) showed compound **1** was consumed completely and desired mass was detected. The crude product (182 mg, crude) was yellow liquid was used into the next step without further purification. To a solution of the crude product (182 mg, 347 μmol, 1.00 *eq*) in PO(OMe)_3_ (1.30 mL) was added (Bu_3_N)_2_H_4_P_2_O_7_ (0.6 M, 2.30 mL, 3.00 *eq*) and Bu_3_N (257 mg, 1388 μmol, 4.00 eq) in at -10 °C. The mixture was stirred at 25 °C for 40 mins. **LCMS** (RT = 0.272 min) showed crude product was consumed completely and one main peak with desired mass was detected. 1.00 M TEAB was added to adjust pH 7. The solution was diluted with H_2_O 2.00 mL and extracted with MTBE (2.00 mL x 3), the aqueous phase purified by DEAE Sephadex column with an elution gradient of 0 to 1.00 M TEAB, evaporated to obtain a colorless oil. The residue was purified by prep-HPLC. Collected and lyophilized. **V2583902** (13.0 mg, 24.0 μmol, 9.75% yield, 97.0% purity) was obtained as a brown solid.

**^1^H NMR:** 400 MHz, D_2_O

δ ppm 8.15 (s, 1H), 7.32 (d, J = 3.60 Hz, 1H), 6.67 (d, J = 4.0 Hz, 1H), 6.44 (d, J = 2.0Hz, 1H), 4.80-4.78 (m, 1H), 4.39-4.29 (m, 3H).

**^31^P NMR:** 162 MHz, D_2_O

δ ppm -10.4--10.1 (1P), -11.9--11.8 (1P), -22.9--22.7 (1P)

**^19^P NMR:** 376 MHz, D_2_O

δ ppm -120.4 (1F)

2′-deoxy-4′-FlA-TP (mCQT310 or V2870202) (Scheme in Figure S17):

Charge cpd **1** (20.0 g, 79.9 mmol 1.00 eq) into (1000 mL bottle) R1. Charge THF (400 mL, 20.0 V) into R1. Charge imidazole (15.56 g, 229 mmol, 2.86 eq) into R1. Charge PPh_3_ (52.4 g, 200mmol, 2.50 eq) into R1. Charge Py (86.6 g, 1.09 mol, 13.7 eq) into R1. Charge I_2_ (40.57 g, 160mmol, 2.00 eq) into R1 at 0 °C. Stir R1 for 4 hrs at 25 °C. Take sample for analysis. (**LCMS**: RT = 0.877 min). The reaction mixture was quenched with sat. aq. Na_2_S_2_O_3_ (400 mL), extracted with EtOAc (400 mL x 2). The organic layer was dried with Na_2_SO_4_, filtered and concentrated under reduced pressure to give a residue. The residue was purified by column chromatography (SiO_2_, petroleum ether/ethyl acetate=1/1 to 0/1 and DCM/MeOH=10/1). Obtain cpd **2** (12.6 g, 35.2 mmol, 43.6% yield) was white solid.

**TLC**: Dichloromethane: Methanol = 5/1, Rf = 0.56

**LCMS:** RT=0.839 min

**^1^H NMR:** 400 MHz, MeOH

δ ppm 8.05 (d, *J* = 6.8 Hz, 1H), 7.35 (d, *J* = 2.8 Hz, 1H), 6.62-6.59 (m, 2H), 4.43-4.40 (m, 1 H), 3.92-3.90 (m, 1H), 3.45-3.36 (m, 2H), 2.66-2.64 (m, 1H), 2.34-2.321 (m, 1H).

Charge cpd **2** (7.30 g, 20.3 mmol, 1.00 eq) into (250 mL bottle) R1. Charge sodium;methanolate (73.0 mL) into R1. Stir R1 for 4 hrs at 60 °C. Take sample for analysis. (**LCMS**: RT = 0.758 min). The reaction solution was added acetic acid to adjust pH 7. The mixture solution was concentrated under reduced pressure to give a residue. The residue was purified by column chromatography (SiO_2_, petroleum ether/ethyl acetate=1/1 to 0/1 and DCM/MeOH=10/1). Obtain cpd **3** (3.00 g, 12.9 mmol, 63.7% yield) was white solid.

**TLC**: Petroleum ether/Ethyl acetate = 0/1, Rf = 0.20

**LCMS**: RT=0.737 min

Charge cpd **3** (2.80 g, 12.1 mmol, 1.00 eq) into (100 mL bottle) R1. Charge ACN (28.0 mL, 10.0 V) into R1. Charge DMAP (0.74 g, 6.03 mmol, 0.05 eq) into R1. Charge Ac_2_O (4.923 g, 48.2 mmol, 4.00 eq) into R1. Stir R1 for 4 hrs at 30 °C. Take sample for analysis. (**LCMS**: RT = 0.911 min). The solution was concentrated under reduced pressure to give a residue. The residue was diluted with ethyl acetate (100 mL) and extracted with H_2_O (100 mL x 2). The combined organic layer was dried over anhydrous Na_2_SO_4_, filtered and concentrated under reduced pressure to give a residue. Combine with MT01765-54. The residue was purified by prep-HPLC (neutral condition: column: Welch Xtimate C18 250 x 70 mm x10 um; mobile phase: [A: H_2_O (10mM NH_4_HCO_3_); B: ACN];B%: 10.00%-46.00%,17.00 min;flow rate: 150.00ml/min). Obtain cpd **4** (1.51 g, 4.77mmol, 34.1% yield) was white solid.

**LCMS**: RT = 0.915 min

**^1^H NMR:** 400 MHz, MeOH

δ ppm 8.52 (s, 1H), 7.48 (m, 1H), 6.96-6.91 (m, 2H), 5.97-5.95 (m, 1 H), 4.46 (d, *J* = 6.8 Hz, 1H), 4.34 (d, *J* = 6.8 Hz, 1H ), 2.99-2.95 (m, 1H), 2.66 (m, 1H), 2.28 (s, 3H), 2.12 (s, 3H).

Charge cpd **4** (1.13 g, 3.57 mmol, 1.00 eq) into (100 mL bottle) R1. Charge THF (25.0 mL 22.1 V) into R1. Charge 3HF.TEA (1.73 g, 10.72 mmol, 3.00 eq) into R1 at 0 °C. Charge NIS (1.45 g, 6.43 mmol, 1.80 eq) into R1 at 0 °C. Stir R1 for 3 hrs at 25 °C. Take sample for analysis. (**LCMS**: RT = 1.051 min). The solution was concentrated under reduced pressure to give a residue. The residue was purified by column chromatography (SiO_2_, petroleum ether/ethyl acetate=5/1 to 0/1). Obtain cpd 5 (1.28 g, 2.77 mmol, 77.5% yield) as yellow oil.

**TLC**: Petroleum ether/Ethyl acetate **=** 0:1, Rf = 0.60

**LCMS:** RT = 1.052 min

Charge cpd **5** (1.28 g, 2.77 mmol, 1.00 eq) into (100 mL bottle) R1. Charge DMF (25.0 mL 19.5 V) into R1. Charge potassium;benzoate (2.22 g, 13.8 mmol, 5.00 eq) into R1. Charge 18-crown-6 (1.47 g, 5.54 mmol, 2.00 eq) into R1under N_2_ protection. The reaction mixture was stirred for 16 hrs at 100 °C. Take sample for analysis. (**LCMS**: RT = 1.143 min). The solution was concentrated under reduced pressure to give a residue. Combine with MT01765-61. The residue was diluted with ethyl acetate (50.0 mL) and extracted with H_2_O (50.0 mL x 2). The combined organic layer, was dried over anhydrous Na_2_SO_4_, filtered and concentrated under reduced pressure to give a residue. The residue was purified by prep-HPLC (neutral condition: column: WePure Biotech XP tC18 250 x 70 x 10 um; mobile phase: [A: H_2_O(10 mM NH_4_HCO_3_);B: ACN];B%: 25.0%-60.0%, 20.00min;flow rate:150.00ml/min). Obtain cpd **6** (0.40 g, 876μmol, 31.6% yield) as yellow solid.

**LCMS**: RT = 1.144 min

Charge cpd **6** (0.40 g, 876 μmol, 1.00 eq) into (100 mL bottle) R1. Charge methanamine (7.7 mL) into R1 under N_2_ protection. The reaction mixture was stirred for 4 hrs at 30 °C. Take sample for analysis. (**LCMS**: RT = 0.544 min). The solution was concentrated under reduced pressure to give a residue. The residue was added MeOH 1mL and the solution was added to 20 mL DCM. Increase the spinning speed of centrifuge to 4500 rpm and spinning for 5 min. Collect solid. The solid was added 5.00 mL ACN. Increase the spinning speed of centrifuge to 4500 rpm and spinning for 5 min. Collect solid. Obtain cpd **7** (0.131 g, 488 μmol, 55.7% yield) as off-white solid.

**LCMS:** RT = 0.819 min

**1H NMR:** 400 MHz MeOH

δ ppm 8.08 (s, 1H),7.29 (d, *J* = 4.0Hz, 1H), 6.73-6.70 (m, 1H), 6.62 (d, *J* = 4.0Hz, 1H), 3.77-3.74 (m, 2H), 2.66-2.59 (m, 2H).

**^19^F NMR:** 376 MHz, MeOH

δ ppm -127.0 (1F)

Charge cpd **7** (0.10 g, 373 μmol, 1.00 eq) into R1 (a thumb flask). Charge proton sponge (80.0 mg, 373μmol, 1.00 eq) into R1 under N_2_ protection. Charge PO(OMe)_3_ (1.00 mL 10.0 V) into R1 under N_2_ protection. Charge POCl_3_ (86.0 mg, 561μmol, 1.50 eq) into R1 at -10 °C under N_2_ protection. Stir R1 for 1 hr at 15 °C. Take sample for analysis. (**LCMS**: RT = 0.422 min). Obtain crude product (0.143 g, crude) as brown liquid.

To a solution of the crude product (0.143 g, 371 μmol 1.00 eq) in PO(OMe)_3_ (1.00 mL) was added (Bu_3_N)_2_H_4_P_2_O_7_ (0.6 M, 1.11 mmol, 1.86 mL, 3.00 eq) at -10 °C under N_2_ protection. Stir R1 for 1 hr at 15 °C. Take sample for analysis. (**LCMS**: RT = 0.338 min). The reaction solution was added TEAB 1mL. The solution was through a DEAE Sephadex column with an elution gradient of 0 to 1.00 M TEAB at 25°C. Fractions of gradient 0.6-0.9 M were collected and concentrated under reduced pressure to give a residue at 30 °C. The residue was purified by prep-HPLC (neutral condition: column: NanoQ-15L 250 x 150 x 15 UM; mobile phase: [A: H_2_O; B: H_2_O (1M TEAB)]; B%: 0.00%-45.00%, 70.00min). Obtain **V2870202** (16.7 mg, 32.8 μmol, 8.88% yield, TEA) as white solid

**LCMS:** RT = 9.043 min

**^1^H NMR:** 400 MHz, D_2_O

δ ppm 8.10 (s, 1H), 7.40 (m, 1H), 6.68 (m, 1H), 5.02-4.97 (m, 1H), 4.31-4.26 (m, 1H), 2.65-2.54 (m, 2H).

**^31^P NMR:** 162 MHz, D_2_O

δ ppm -10.5--10.6 (1P), -11.7--11.9 (1P), -23.9--23.2(1P)

**^19^F NMR:** 376 MHz, D_2_O

δ ppm -123.5 (1F)

**Table S1**. Selectivity values for 4′-FlA-TP against viral RdRp.

|  | **ATP** | | | **4′-FlA-TP** | | |  |
| --- | --- | --- | --- | --- | --- | --- | --- |
| Virus | V_max_^a^  (product fraction) | *K*_m_^b^ (µM) | Efficiency  (V_max_/*K*_m_) | V_max_  (product fraction) | *K*_m_ (µM) | Efficiency  (V_max_/*K*_m_) | Selectivity^c^ (fold) |
| **DENV-2** | n=4^d^ |  |  |  |  |  |  |
|  | 0.91 | 0.12 | 7.6 | 0.85 | 0.84 | 1.0 | 7.6 |
| **±**^e^ | 0.01 | 0.01 |  | 0.01 | 0.05 |  |  |
| **HRV-16** | n=3 |  |  |  |  |  |  |
|  | 0.79 | 1.5 | 0.53 | 0.75 | 0.70 | 1.1 | 0.48 |
| **±** | 0.02 | 0.2 |  | 0.02 | 0.07 |  |  |
| **SARS-COV-2** | n=3 |  |  |  |  |  |  |
|  | 0.69 | 0.070 | 9.9 | 0.54 | 0.053 | 10 | 0.99 |
| **±** | 0.01 | 0.003 |  | 0.01 | 0.002 |  |  |
| **RSV** | n=3 |  |  |  |  |  |  |
|  | 0.72 | 0.69 | 1.0 | 0.49 | 3.5 | 0.14 | 7.1 |
| **±** | 0.02 | 0.11 |  | 0.02 | 0.5 |  |  |
| **EBOV** | n=3 |  |  |  |  |  |  |
|  | 0.63 | 1.9 | 0.33 | 0.52 | 6.6 | 0.079 | 4.2 |
| **±** | 0.04 | 0.4 |  | 0.03 | 1.4 |  |  |
| **CCHFV** | n=3 |  |  |  |  |  |  |
|  | 0.62 | 0.26 | 2.4 | 0.57 | 0.33 | 1.7 | 1.4 |
| **±** | 0.03 | 0.06 |  | 0.04 | 0.09 |  |  |
| **FluB** | n=4 |  |  |  |  |  |  |
|  | 0.80 | 0.19 | 4.2 | 0.80 | 0.26 | 3.1 | 1.4 |
| **±** | 0.02 | 0.02 |  | 0.02 | 0.04 |  |  |

^a^ V_max_ is a Michaelis–Menten parameter reflecting the maximal velocity of nucleotide incorporation.

^b^ *K*_m_ is a Michaelis–Menten parameter reflecting the concentration of the nucleotide substrate at which the velocity of nucleotide incorporation is half of V_max_.

^c^ Selectivity of a viral RNA polymerase for a nucleotide substrate analog is calculated as the ratio of the V_max_/*K_m_* values for NTP and NTP analog, respectively.

^d^ All reported values have been calculated based on an 8–data point experiment repeated the indicated number of times (n).

^e^ Standard error associated with the fit.

**Table S2**. Selectivity values for RDV-TP and 4′-FlA-TP against influenza RdRp using 13-nt capped primer system.

|  | Nucleotide | V_max_^a^  (product fraction) | *K*_m_^b^ (µM) | Efficiency  (V_max_/*K*_m_) | Selectivity^c^ (fold) |
| --- | --- | --- | --- | --- | --- |
| FluA H3N2  (n=3)^d^ | ATP | 0.43 ±^e^ 0.02 | 0.034 ± 0.007 | 13 | **Ref.**^f^ |
|  | 4′-FlA-TP | 0.39 ± 0.03 | 0.046 ± 0.013 | 8.5 | 1.5 |
|  | RDV-TP | 0.39 ± 0.02 | 57 ± 8 | 0.0068 | 1800 |
|  |  | | | | |
| FluB  (n=3) | ATP | 0.50 ± 0.03 | 0.060 ± 0.013 | 8.3 | **Ref.** |
|  | 4′-FlA-TP | 0.53 ± 0.03 | 0.033 ± 0.006 | 16 | 0.52 |
|  | RDV-TP | 0.19 ± 0.01 | 32 ± 8 | 0.0059 | 1400 |

^a^ V_max_ is a Michaelis–Menten parameter reflecting the maximal velocity of nucleotide incorporation.

^b^ *K*_m_ is a Michaelis–Menten parameter reflecting the concentration of the nucleotide substrate at which the velocity of nucleotide incorporation is half of V_max_.

^c^ Selectivity of a viral RNA polymerase for a nucleotide substrate analog is calculated as the ratio of the V_max_/*K_m_* values for NTP and NTP analog, respectively.

^d^ All reported values have been calculated based on an 8–data point experiment repeated the indicated number of times (n).

^e^ Standard error associated with the fit.

^f^ Reference.

**Table S3**. Inhibitory effect of the incorporated analog-monophosphate on the incorporation of the subsequent nucleotide.

| Enzyme | Primer 3’-end (base) | Substrate | V_max_c (product fraction) | K_m_^d^ (µM) | V_max_/K_m_ | Inhibition^e^ (fold) |
| --- | --- | --- | --- | --- | --- | --- |
| **DENV-2**  (n=3)^f^ | AMP^a^ (1 µM)^b^ | UTP | 0.84 ±^g^ 0.03 | 0.060 ± 0.008 | 14 | Ref.^h^ |
|  | 4′-FlA-MP (10 µM) |  | 0.84 ± 0.03 | 0.23 ± 0.02 | 3.7 | 3.8 |
| **HRV-16**  (n=3) | AMP (10 µM) | UTP | 0.60 ± 0.01 | 0.044 ± 0.004 | 14 | Ref. |
|  | 4′-FlA-MP (10 µM) |  | 0.62 ± 0.02 | 0.13 ± 0.01 | 4.8 | 2.9 |
| **SARS-CoV-2**  (n=4) | AMP (1 µM) | UTP | 0.70 ± 0.01 | 0.013 ± 0.001 | 54 | Ref. |
|  | 4′-FlA-MP (1 µM) |  | 0.68 ± 0.02 | 0.068 ± 0.008 | 10 | 5.4 |
| **RSV**  (n=3) | AMP (100 µM) | UTP | 0.61 ± 0.03 | 0.69 ± 0.10 | 0.88 | Ref. |
|  | 4′-FlA-MP (100 µM) |  | 0.58 ± 0.02 | 8.8 ± 1.0 | 0.066 | 13 |
| **EBOV**  (n=4) | AMP (100 µM) | UTP | 0.31 ± 0.02 | 4.0 ± 1.0 | 0.078 | Ref. |
|  | 4′-FlA-MP (100 µM) |  | 0.29 ± 0.01 | 30 ± 5.2 | 0.0097 | 8.0 |
| **CCHFV**  (n=3) | AMP (10 µM) | UTP | 0.63 ± 0.02 | 0.20 ± 0.04 | 3.2 | Ref. |
|  | 4′-FlA-MP (10 µM) |  | 0.43 ± 0.02 | 4.4 ± 0.6 | 0.098 | 33 |
| **FluB**  (n=3) | AMP (10 µM) | UTP | 0.69 ± 0.02 | 0.012 ± 0.001 | 58 | Ref. |
|  | 4′-FlA-MP (10 µM) |  | 0.42 ± 0.01 | 5.1 ± 0.5 | 0.082 | 710 |
|  |  |  |  |  |  |  |
| **h-mtRNAP**  (n=3) | AMP (1 µM) | CTP | 0.36 ± 0.01 | 0.033 ± 0.002 | 11 | Ref. |
|  | 4′-FlA-MP (10 µM) |  | 0.32 ± 0.01 | 0.053 ± 0.004 | 6.0 | 1.8^i^ |

^a^ Denotes that the 3´-end of the primer is terminated by the monophosphate form of the respective nucleotide.

^b^ Concentration of the respective nucleotide that terminates the 3´-end of the primer.

^c^ V_max_ is a Michaelis–Menten parameter reflecting the maximal velocity of nucleotide incorporation.

^d^ *K*_m_ is a Michaelis–Menten parameter reflecting the concentration of the nucleotide substrate at which the velocity

of nucleotide incorporation is half of Vmax.

^e^ Inhibition of subsequent nucleotide incorporation is calculated as the ratio of Vmax/*K*m values for UTP determined

on primers terminated with AMP or 4′-FlA.

^f^ All reported values have been calculated based on an 8–data point experiment repeated the indicated number of times (n).

^g^ Standard error associated with the fit.

^h^ Reference.

^i^ Inhibition of subsequent nucleotide incorporation is calculated as the ratio of Vmax/*K*m values for CTP determined

on primers terminated with AMP or 4′-FlA.

**Table S4**. Cryo-EM data collection, refinement, and validation statistics for SARS-CoV-2 RdRp complex with dsRNA and 4′-FlA-TP

| **Data Collection and Processing** | |
| --- | --- |
| Electron microscope | TFS Glacios 2 |
| Electron detector | Falcon 4i |
| Magnification | 190,000x |
| Voltage (kV) | 200 |
| Electron exposure (e^-^/Å^2^) | 45 |
| Defocus range (μm) | -0.8 to -1.8 |
| Pixel Size (Å) | 0.718 |
| Symmetry imposed | C1 |
| Number of movie micrographs | 7,824 |
| Number of particle images in map | 48,482 |
| Map resolution (Å) | 3.38 |
| FSC threshold | 0.143 |
| Map sharpening *B* factor (Å^2^) | -78.8 |
| **Model building and validation** | |
| *Model composition* | |
| Protein Chains | 4 |
| Protein Residues | 1,176 |
| RNA Chains | 2 |
| RNA Residues | 23 |
| 4′-FlA molecules | 1 |
| *RMSD bonds* | |
| Bond lengths (Å) | 0.016 |
| Bond angles (°) | 0.838 |
| *Ramachandran plot* | |
| Favored (%) | 97.84 |
| Outliers (%) | 0.00 |
| *Validation* | |
| MolProbity score | 1.34 |
| Clashscore | 5.51 |
| Rotamer outliers (%) | 0.58 |
| EMRinger score | 2.50 |
| Map-model correlation coefficient, CC (mask) | 0.78 |

**Table S5**. Selectivity values for nucleotide analogs against off-target polymerases.

|  | Nucleotide | V_max_^a^  (product fraction) | *K*_m_^b^ (µM) | Efficiency  (V_max_/*K*_m_) | Selectivity^c^ (fold) |
| --- | --- | --- | --- | --- | --- |
| h-mtRNAP | ATP | 0.39^d^ ±^e^ 0.01 | 0.015 ± 0.001 | 26 | **Ref.**^f^ |
|  | RDV-TP | 0.14 ± 0.010 | 12 ± 4 | 0.012 | 2,200 |
|  | 4′-FlA-TP | 0.43 ± 0.004 | 0.14 ± 0.01 | 3.1 | 8.4 |
|  |  | | | | |
|  | UTP | 0.38 ± 0.01 | 0.029 ± 0.002 | 13 | **Ref.** |
|  | SOF-TP |  |  |  | B.L.Q. ^g^ |
|  | 4′-FlU-TP | 0.48 ± 0.01 | 0.15 ± 0.02 | 3.2 | 4.1 |
|  |  |  |  |  |  |
|  | CTP | 0.46 ± 0.01 | 0.039 ± 0.003 | 12 | **Ref.** |
|  | NHC-TP | 0.59 ± 0.03 | 1.1 ± 0.3 | 0.54 | 22 |
|  |  |  |  |  |  |
| Pol γ | dATP | 0.37 ± 0.03 | 0.012 ± 0.003 | 31 | **Ref.** |
|  | ddATP | 0.28 ± 0.02 | 0.0060 ± 0.0012 | 47 | 0.66 |
|  | TFV-DP | 0.29 ± 0.01 | 2.0 ± 0.3 | 0.15 | 210 |
|  | 2′-d-4′-FlA-TP | 0.36 ± 0.02 | 0.010 ± 0.003 | 33 | 0.94 |
|  |  |  |  |  |  |
| Klenow  -exonuclease (KF) | dATP | 0.38 ± 0.01 | 0.025 ± 0.002 | 15 | **Ref.** |
|  | ddATP | 0.22 ± 0.02 | 11 ± 4 | 0.020 | 750 |
|  | TFV-DP |  |  |  | B.L.Q. |
|  | 2′-d-4′-FlA-TP | 0.29 ± 0.02 | 0.094 ± 0.020 | 3.1 | 4.8 |

^a^ V_max_ is a Michaelis–Menten parameter reflecting the maximal velocity of nucleotide incorporation.

^b^ *K*_m_ is a Michaelis–Menten parameter reflecting the concentration of the nucleotide substrate at which the velocity of nucleotide incorporation is half of V_max_.

^c^ Selectivity of a viral RNA polymerase for a nucleotide substrate analog is calculated as the ratio of the V_max_/*K_m_* values for NTP and NTP analog, respectively.

^d^ All reported values have been calculated based on an 8–data point experiment repeated at least 3 times (n=3).

^e^ Standard error associated with the fit.

^f^ Reference.

^g^ Below the level of quantification, B.L.Q.

**Figure S1. Selective incorporation of 4′-FlA-TP by RSV RdRp.** (**A**) RNA primer/template substrate used in the RNA synthesis assays to evaluate 4′-FlA-TP as an A-analog substrate is shown above the gel. G indicates incorporation of the radiolabelled nucleotide opposite template position 5. Position (“i”) allows incorporation of ATP or 4′-FlA-TP. (**B**) NTP incorporation was monitored with purified RSV RdRp complex in the presence of [α-^32^P]-GTP, RNA primer/template, MgCl_2_, and increasing concentrations of ATP or 4′-FlA-TP. Lane m illustrates the migration pattern of the radiolabelled 4-nucleotide-long primer. (**C**) Graphic representation of the data for incorporation of ATP and 4′-FlA-TP. (**D**) Kinetic parameters and efficiency of ATP and 4′-FlA-TP incorporation by RSV RdRp. Efficiency is determined as the quotient of V_max_ over *K*_m_. Selectivity is the quotient of ATP efficiency over ATP-analog efficiency.

**Figure S2. Selective incorporation of 4′-FlA-TP by FluA RdRp in the capped primer extension assay.** (**A**) 3′ vRNA template and 13-nt capped radiolabeled RNA primer used to evaluate A-analog incorporation at position 14 (“i”). (**B**) Nucleotide incorporation was monitored with purified FluA RdRp complex in the presence of the radiolabelled RNA primer, vRNA template, MgCl_2,_ BXA (to prevent endonuclease cleavage of the capped primer), and increasing concentrations of the indicated nucleotide (ATP, 4′-FlA-TP, or RDV-TP). Lane m indicates the 13-nt labelled primer used as a marker. (**C**) Graphic representation of ATP and 4′-FlA-TP incorporation. (**D**) Kinetic parameters determined from quantification of product signal, which is ATP or 4′-FlA-TP incorporation by FluA RdRp. Efficiency is determined as the quotient of V_max_ over *K*_m_. Selectivity is the quotient of ATP efficiency over ATP-analog efficiency.

**Figure S3. HRV-16 and SARS-CoV-2 RdRp-catalyzed RNA synthesis and inhibition patterns following a single incorporation of ATP or 4′-FlA-TP as a function of nucleotide concentration.** (**A**) RNA primer/template supporting a single incorporation of ATP or 4′-FlA-TP at position 6 (“i”). G indicates incorporation of [α-^32^P]-GTP at position 5. Migration pattern of RNA products resulting from (**B**) HRV-16 RdRp- and (**C**) SARS-CoV-2 RdRp-catalyzed RNA extension following incorporation of ATP or 4′-FlA-TP at position 6 (“i”) at increasing CTP and UTP concentrations. A 5′-^32^P-labelled 4-nt primer (4) serves as a size marker (m). RNA products formed at and beyond the asterisk may be a result of sequence-dependent slippage events or RdRp nucleotide transferase activity. HRV-16, human rhinovirus 16; SARS-CoV-2, severe acute respiratory syndrome coronavirus 2; RdRp, RNA-dependent RNA polymerase.

**Figure S4. CCHFV RdRp-catalyzed RNA synthesis and inhibition patterns following a single incorporation of ATP or 4′-FlA-TP as a function of nucleotide concentration.** (**A**) RNA primer/template supporting a single incorporation of ATP or 4′-FlA-TP at position 6 (“i”). G indicates incorporation of [α-^32^P]-GTP at position 5. Migration pattern of RNA products resulting from (**B**) CCHFV RdRp-catalyzed RNA extension following incorporation of ATP or 4′-FlA-TP at position 6 (“i”) at increasing CTP and UTP concentrations. A 5′-^32^P-labelled 4-nt primer (4) serves as a size marker (m). CCHFV, Crimean-Congo hemorrhagic fever virus; RdRp, RNA-dependent RNA polymerase.

**Figure S5. RSV RdRp-catalyzed RNA synthesis and inhibition patterns following a single or multiple incorporations of ATP or 4′-FlA-TP as a function of nucleotide concentration.** (**A**) RNA primer/template supporting a single incorporation of ATP or 4′-FlA-TP at position 6 (“i”). G indicates incorporation of [α-32P]-GTP at position 5. Migration pattern of RNA products resulting from RSV RdRp-catalyzed RNA extension following a single incorporation of ATP or 4′-FlA-TP at increasing CTP and UTP concentrations. Investigation of a single incorporated 4′-FlA-TP is difficult, given that full-template length product is not generated even under the natural scenario with ATP, which has been observed previously (1). A 5′-^32^P-labelled 4-nt primer (4) serves as a size marker (m). (**B**) RNA primer/template supporting a first and second incorporation of ATP or 4′-FlA-TP at position 6 (“i”) and position 9 (“i_2_”). G indicates incorporation of [α-^32^P]-GTP at position 5. Migration pattern of RNA products resulting from RSV RdRp-catalyzed RNA extension following multiple incorporations of ATP or 4′-FlA-TP at increasing CTP and UTP concentrations. A 5′-^32^P-labelled 4-nt primer (4) serves as a size marker (m). Multiple 4′-FlA-TP incorporations do not appear to allow production of full-template length product. RSV, Respiratory syncytial virus; RdRp, RNA-dependent RNA polymerase.

**Figure S6. FluA and FluB RdRp-catalyzed RNA synthesis and inhibition patterns following multiple incorporations of ATP or 4′-FlA-TP as a function of nucleotide concentration.** (**A**) A 13-nt capped radiolabeled RNA primer and vRNA template required for FluA RdRp activity. Reactions consist of vRNA template, capped primer, viral RdRp, BXA (an influenza endonuclease inhibitor), ATP or 4′-FlA-TP, increasing concentrations of CTP and GTP, and Mg^2+^ (which starts the reaction). The primer-template supports the incorporation of ATP or 4′-FlA-TP at position 14 (“i”). Additional incorporations of ATP or 4′-FlA-TP can occur as indicated. Migration pattern of RNA products resulting from (**B**) FluA RdRp- and (**C**) FluB RdRp-catalyzed RNA extension following incorporation of ATP or 4′-FlA-TP at position 14 (“i”) at increasing CTP and GTP concentrations. 5′-^32^P-labelled 13-nt primer (13) serves as a size marker (m). Investigation of 4′-FlA-TP using this template is difficult given a single incorporation event is not possible. A second site of 4′-FlA-TP incorporation does not appear to be overcome. FluA, Influenza A virus; FluB, Influenza B virus; RdRp, RNA-dependent RNA polymerase.

**Figure S7. Inhibitory effect of RNA extension by CCHFV RdRp following 4′-FlA-TP incorporation.** (**A**) RNA primer/template supporting a single incorporation of ATP or 4′-FlA-TP at position 6 (“i”) and a subsequent incorporation of UTP at position 7 (“i+1”). G indicates incorporation of [α-^32^P]-GTP at position 5. (**B**) CCHFV RdRp-catalyzed RNA extension to position “i+1” following incorporation of ATP or 4′-FlA-TP at position 6 (“i”) at increasing UTP concentrations. ATP or 4′-FlA-TP were kept at fixed concentrations. RNA products were resolved by denaturing PAGE. A 5′-^32^P-labelled 4-nt primer (4) serves as a size marker (m). (**C**) Graphic representation of the data for incorporation of UTP by CCHFV RdRp following the incorporation of ATP and 4′-FlA-TP. (**D**) Kinetic parameters and efficiency of UTP incorporation by CCHFV RdRp following the incorporation of ATP and 4′-FlA-TP. Efficiency is determined as the quotient of V_max_ over *K*_m_. Inhibition is the quotient of UTP incorporation efficiency following ATP incorporation, divided by the efficiency of UTP incorporation following 4′-FlA-TP incorporation. CCHFV, Crimean-Congo hemorrhagic fever virus; RdRp, RNA-dependent RNA polymerase.

**Figure S8. Template-dependent inhibition of HRV-16, SARS-CoV-2, and CCHFV RdRp by a single embedded 4′-FlA residue at position 11.** (**A**) RNA primer/template with an embedded adenosine (Template “A”, *left*) and (Template “F”, *right*) position 11. G indicates incorporation of [α-^32^P]-GTP at position 5. Migration pattern of RNA products resulting from (**B**) HRV-16 RdRp-, (**C**) SARS-CoV-2 RdRp-, (**D**) and CCHFV RdRp-catalyzed RNA synthesis supplemented with increasing NTP concentrations using Template “A” (*left*) and Template “F” (*right*). A 5′-^32^P-labelled 4-nt primer (4) serves as a size marker (m). RNA products formed at and beyond the asterisk indicate slippage products that may be a result of sequence-dependent slippage events or RdRp nucleotide transferase activity. HRV, human rhinovirus; SARS-CoV-2, severe acute respiratory syndrome coronavirus 2; CCHFV, Crimean-Congo hemorrhagic fever virus; RdRp, RNA-dependent RNA polymerase.

**Figure S9. Impairment of NTP incorporation opposite a template-embedded 4′-FlA.** (**A**) RNA primer and RNA template with either an embedded adenosine (Template “A”) or (Template “F”) at position 11 (indicated as “X” in the diagram). G indicates incorporation of [α-^32^P]-GTP at position 5. *Step 1*: DENV-2 RdRp, [α-^32^P]-GTP, ATP, and MgCl_2_ are added to the reaction mixture, allowing for RNA synthesis up to position 11. After 10 minutes, the first zero lane reaction (“Initial zero”) is withdrawn. *Step 2*: After the 10 minutes, increasing concentrations (0.1, 1, 10, 100, 1000 µM) of the various NTPs are added to the reaction to allow for incorporation at and beyond position 11. A second zero (“0”) has no NTP added. After 10 minutes, reactions are stopped with formamide/EDTA mixture. (**B**) Migration pattern of RNA products resulting from DENV-2 RdRp-catalyzed RNA synthesis with increasing NTP concentrations using Template “A” (*left*) and Template “F” (*right*). A 5′-^32^P-labelled 4-nt primer (4) serves as a size marker (m). RNA products formed at and beyond the asterisk may be a result of sequence-dependent slippage events or RdRp nucleotide transferase activity. (**C**) Graphic representation of product fraction on Template “F” as a function of NTP concentration. Product fraction is defined as RNA products generated beyond position 10 in each lane, divided by the total signal in that lane, normalized to the UTP lane with the highest product fraction. (**D**) Graphic representation of product fraction on Template “A” and Template “F” as a function of NTP concentration. Product fraction is defined as RNA products generated beyond position 10 in each lane, divided by the total signal in that lane, normalized to the UTP lane on Template “**A**” with the highest product fraction. DENV-2, dengue virus 2; RdRp, RNA-dependent RNA polymerase.

**Figure S10. Structural basis for 4′-FlA incorporation by the SARS-CoV-2 RdRp and cryo-EM data quality.** (**A**) Structural superposition of the 4′-FlA-bound RdRp complex (purple) with the remdesivir (RDV)-bound RdRp structure (white). Both 4′-FlA and RDV are shown as stick models and are indicated by arrows. (**B**) Cryo-EM density map of 4′-FlA molecule incorporated at the +1 position and the adjacent -1 nucleotide of the primer strand. The map is shown as a mesh, and nucleotides are displayed as stick models. (**C**) Local resolution map of the cryo-EM density. (**D**) Gold-standard Fourier shell correlation (GSFSC) curve (top). Angular distribution plot of particles used for the final reconstruction (bottom). (**E**) Structural superposition of 4′-FlA resolved in this study with DENV-2 RdRp elongation complex. (**F**) Structural superposition of 4′-FlA resolved in this study with FluB RdRp elongation complex. (**G** and **H**) Modelled interactions between the 4′-fluoro modification and nsp12 residues in the primer strand (**G**) and template strand (**H**), based on PDB: 7BV2 (2). RNA nucleotides are shown as green (template) or cyan (primer) sticks, with the 4′-fluorine atom displayed as a green sphere. All contacts within 3.5 Å of the fluorine atom are indicated by dashed lines, with contacts shorter than 3.0 Å shown as red dashed lines with distances labelled. For backbone interactions, the relevant atoms are explicitly displayed. Interacting nsp12 residues are labelled.

**Figure 11. Chemical structures of additional nucleoside and nucleotide analogs investigated in this study.** The active form of molnupiravir or NHC-TP (**A**), the active form of sofosbuvir or SOF-TP (**B**), the active form of 4′-FlU or 4′-FlU-TP (**C**), the active form of tenofovir or TFV-DP (**D**), ddATP (**E**), and 2′-deoxy-4′-FlA-TP (**F**).

**Figure S12. Incorporation of nucleotide analogs by h-mtRNAP.** (**A**) RNA primer/DNA template substrate (*Top*) used in the RNA synthesis assays to evaluate RDV-TP and 4′-FlA-TP as a substrate for incorporation as an A-analog. Position 13 indicates the length of the Cy5-labelled primer. Position 14 (“i”) allows incorporation of ATP, RDV-TP, or 4′-FlA-TP. (*Bottom*) NTP incorporation was monitored with purified h-mtRNAP DdRp complex in the presence of RNA primer/DNA template, MgCl_2_, and increasing concentrations of ATP, RDV-TP, or 4′-FlA-TP. Lane m illustrates the migration pattern of the Cy5-labelled 13-nucleotide-long primer. (**B**) (*Top*) Graphic representation of the data for incorporation of ATP, RDV-TP, and 4′-FlA-TP. (*Bottom*) Kinetic parameters and efficiency of ATP, RDV-TP, and 4′-FlA-TP incorporation by h-mtRNAP DdRp. Efficiency is determined as the quotient of V_max_ over *K*_m_. Selectivity is the quotient of ATP efficiency over ATP-analog efficiency. (**C**) RNA primer/DNA template substrate (*Top*) used in the RNA synthesis assays to evaluate SOF-TP and 4′-FlU-TP as a substrate for incorporation. Position 13 indicates the length of the Cy5-labelled primer. Position 14 (“i”) allows incorporation of UTP, SOF-TP, or 4′-FlU-TP. (*Bottom*) NTP incorporation was monitored with purified h-mtRNAP DdRp complex in the presence of RNA primer/DNA template, MgCl_2_, and increasing concentrations of UTP, SOF-TP, or 4′-FlU-TP. Lane m illustrates the migration pattern of the Cy5-labelled 13-nucleotide-long primer. (**D**) (*Top*) Graphic representation of the data for incorporation of UTP, SOF-TP, and 4′-FlU-TP. (*Bottom*) Kinetic parameters and efficiency of UTP, SOF-TP, and 4′-FlU-TP incorporation by h-mtRNAP DdRp. Efficiency is determined as the quotient of V_max_ over *K*_m_. Selectivity is the quotient of UTP efficiency over UTP-analog efficiency. (**E**) RNA primer/DNA template substrate (*Top*) used in the RNA synthesis assays to evaluate NHC-TP as a substrate for incorporation. Position 13 indicates the length of the FAM-labelled primer. Position 14 (“i”) allows incorporation of CTP or NHC-TP. (*Bottom*) NTP incorporation was monitored with purified h-mtRNAP DdRp complex in the presence of RNA primer/DNA template, MgCl_2_, and increasing concentrations of CTP or NHC-TP. Lane m illustrates the migration pattern of the FAM-labelled 13-nucleotide-long primer. (**F**) (*Top*) Graphic representation of the data for incorporation of CTP and NHC-TP. (*Bottom*) Kinetic parameters and efficiency of CTP and NHC-TP incorporation by h-mtRNAP DdRp. Efficiency is determined as the quotient of V_max_ over *K*_m_. Selectivity is the quotient of UTP efficiency over UTP-analog efficiency. Below the level of quantification, B.L.Q.

**Figure S13. Modelled binding of 4′-FlA-TP in the human mitochondrial RNA polymerase (h-mtRNAP) initiation catalytic complex.** (**A**) Structure of the h-mtRNAP initiation complex (PDB: 9R96) (3), which is composed of h-mtRNAP (blue), transcription factor TFB2M (yellow), non-template (cyan), template (pink), and incoming GTP (green). (**B**) The nucleotide analog 4′-FlA-TP is modelled at the N-site within h-mtRNAP. Metal A and B are represented with pink spheres. (**C**) The 4′-fluoro group of 4′-FlA-TP fits well within the adjacent molecular surface of h-mtRNAP. (**D**) In contrast to 4′-FlA-TP, the 1′-cyano group of RDV-TP appears to clash with h-mtRNAP, suggesting it is a poor substrate.

**Figure S14. Incorporation of nucleotide analogs by Pol γ.** (**A**) DNA primer/template substrate used in the DNA synthesis assays for Pol γ and KF to evaluate ddATP, TFV-DP, and 2′-deoxy-4′-FlA-TP as a substrate for incorporation as an A-analog incorporation is shown above the gel. Position (“i”) allows incorporation of dATP, ddATP, TFV-DP, or 2′-deoxy-4′-FlA-TP. (**B**) Nucleotide incorporation was monitored with purified Pol γ DdDp complex in the presence of Cy5-labelled primer annealed to DNA template, MgCl_2_, and increasing concentrations of dATP, ddATP, TFV-DP, or 2′-deoxy-4′-FlA-TP. Lane m illustrates the migration pattern of the Cy5-labelled primer used as a marker. (**C**) Graphic representation of dATP, ddATP, TFV-DP, and 2′-deoxy-4′-FlA-TP incorporation. (**D**) Kinetic parameters and efficiency of dATP, ddATP, TFV-DP, and 2′-deoxy-4′-FlA-TP incorporation by Pol γ DdDp. Efficiency is determined as the quotient of V_max_ over *K*_m_. Selectivity is the quotient of ATP efficiency over ATP-analog efficiency.

**Figure S15. DNA Pol 𝛾 and Klenow DdDp-catalyzed DNA synthesis and inhibition patterns following a single incorporation of dATP or 2′-d-4′-FlA-TP as a function of nucleotide concentration.** (**A**) DNA primer/template supporting a single incorporation of dATP or 2′-d-4′-FlA-TP at position 21 (“i”). The DNA primer is 5′-Cy5 labelled for signal detection of synthesized DNA products. (**B**) Migration pattern of DNA products resulting from DNA Pol 𝛾 DdDp-catalyzed extension following incorporation of dATP, ddATP, or 2′-d-4′-FlA-TP at position 14 (“i”) at increasing concentrations of dCTP, dGTP, and dTTP. (**C**) Migration pattern of DNA products resulting from Klenow DdDp-catalyzed extension following incorporation of dATP or 2′-d-4′-FlA-TP at position 14 (“i”) at increasing concentrations of dCTP, dGTP, and dTTP.

**Figure S16.** Chemical synthesis scheme of 4′-fluoroadenosine triphosphate (4′-FlA-TP).

**Figure S17.** Chemical synthesis scheme of 2′-deoxy-4′-fluoroadenosine triphosphate (2′-d-4′-FlA-TP).

**References**

Ref. 40 in manuscript

1.	Tchesnokov, E.P., Feng, J.Y., Porter, D.P. and Gotte, M. Mechanism of inhibition of Ebola virus RNA-dependent RNA polymerase by remdesivir. *Viruses*, 2019; **11**, 326. 10.3390/v11040326

Ref. 42 in manuscript

2.	Yin, W., Mao, C., Luan, X., Shen, D.D., Shen, Q., Su, H., Wang, X., Zhou, F., Zhao, W., Gao, M. *et al.* Structural basis for inhibition of the RNA-dependent RNA polymerase from SARS-CoV-2 by remdesivir. *Science*, 2020; **368**: 1499-1504. 10.1126/science.abc1560

Ref. 58 in manuscript

3.	Shen, J., Goovaerts, Q., Ajjugal, Y., De Wijngaert, B., Das, K. and Patel, S.S. Human mitochondrial RNA polymerase structures reveal transcription start site and slippage mechanism. *Mol Cell*, 2025; **85**: 3137-3150 e3137. 10.1016/j.molcel.2025.07.002
